# An Extensive Knowledge Mapping Review of Measurement and Validity in Language Assessment and SLA Research

**DOI:** 10.3389/fpsyg.2020.01941

**Published:** 2020-09-04

**Authors:** Vahid Aryadoust, Azrifah Zakaria, Mei Hui Lim, Chaomei Chen

**Affiliations:** ^1^National Institute of Education, Nanyang Technological University, Singapore, Singapore; ^2^Nanyang Technological University, Singapore, Singapore; ^3^College of Computing and Informatics, Drexel University, Philadelphia, PA, United States; ^4^Department of Information Science, Yonsei University, Seoul, South Korea

**Keywords:** document co-citation analysis, language assessment, measurement, review, Scientometrics, validity, visualization, Second language acquisition

## Abstract

This study set out to investigate intellectual domains as well as the use of measurement and validation methods in language assessment research and second language acquisition (SLA) published in English in peer-reviewed journals. Using Scopus, we created two datasets: (i) a dataset of core journals consisting of 1,561 articles published in four language assessment journals, and (ii) a dataset of general journals consisting of 3,175 articles on language assessment published in the top journals of SLA and applied linguistics. We applied document co-citation analysis to detect thematically distinct research clusters. Next, we coded citing papers in each cluster based on an analytical framework for measurement and validation. We found that the focus of the core journals was more exclusively on reading and listening comprehension assessment (primary), facets of speaking and writing performance such as raters and validation (secondary), as well as feedback, corpus linguistics, and washback (tertiary). By contrast, the primary focus of assessment research in the general journals was on vocabulary, oral proficiency, essay writing, grammar, and reading. The secondary focus was on affective schemata, awareness, memory, language proficiency, explicit vs. implicit language knowledge, language or semantic awareness, and semantic complexity. With the exception of language proficiency, this second area of focus was absent in the core journals. It was further found that the majority of citing publications in the two datasets did not carry out inference-based validation on their instruments before using them. More research is needed to determine what motivates authors to select and investigate a topic, how thoroughly they cite past research, and what internal (within a field) and external (between fields) factors lead to the sustainability of a Research Topic in language assessment.

## Introduction

Although the practice of language testing and/or assessment can be traced back in history to ancient eras in China (Spolsky, 1990), many language assessment scholars recognize the pioneering book of Lado's (1961) and the book chapter of Carroll's (1961), as the beginning of the modern language testing/assessment field (Davies, 2008, 2014). The field was routinely referred to as language testing, at least from the 1950s until the 1990s. In contemporary usage, it is possible to make a distinction between testing and assessment, in terms of the formality and stakes involved in the procedures, the use of quantitative vs. qualitative approaches in design and implementation and other aspects^1^. Nonetheless, in the present study, testing, and assessment are used interchangeably. Despite the general recognition of 1961 as the beginning of the field of language testing, there had been many language testing studies published before 1961, particularly in the field of reading (e.g., Langsam, 1941; Davis, 1944; Hall and Robinson, 1945; see also Rosenshine, 2017; Aryadoust, 2020 for reviews). By definition, these studies qualify as language testing research and practice since they meet several criteria that Priscilla Allen, Alan Davies, Carol Chapelle and Geoff Brindley, and F. Y. Edgeworth set forth in their delineations of language testing, most notably the practice of evaluating language ability/proficiency, the psychometric activity of developing language tests, and/or decision making about test takers based on test results (Fulcher, n.d.).

In order to build a fair portrayal of a discipline, researchers often review the research outputs that have been generated over the years to understand its past and present trends (Goswami and Agrawal, 2019). For language assessment, several scholars have surveyed the literature and divided its development into distinct periods (Spolsky, 1977, 1995; Weir, 1990; Davies, 2014), while characterizing its historical events (Spolsky, 2017). Alternatively, some provided valuable personal reflections on the published literature (Davies, 1982; Skehan, 1988; Bachman, 2000; Alderson and Banerjee, 2001, 2002). Examples of personal reflections on specific parts of language assessment history also include Spolsky's (1990) paper on the “prehistory” of oral examinations and Weir et al.'s (2013) historical review of Cambridge assessments.

These narrative reviews offer several advantages such as the provision of “experts' intuitive, experiential, and explicit perspectives on focused topics” (Pae, 2015, p. 417). On the other hand, narrative reviews are qualitative in nature and do not use databases or vigorous frameworks and methodologies (Jones, 2004; Petticrew and Roberts, 2006). This contrasts with quantitative reviews, which have specific research questions or hypotheses and rely on the quantitative evaluation and analysis of data (Collins and Fauser, 2005). An example of such an approach is Scientometrics which is “the quantitative methods of the research on the development of science as an informational process” (Nalimov and Mulcjenko, 1971, p. 2). This approach comprises several main themes including “ways of measuring research quality and impact, understanding the processes of citations, mapping scientific fields and the use of indicators in research policy and management” (Mingers and Leydesdorff, 2015, p. 1). This wide scope makes Scientometrics a specialized and “extensively institutionalized area of inquiry” (De Bellis, 2014, p. 24). Thus, it is appropriate for analyzing the entire areas of research across various research fields (Mostafa, 2020).

### Present Study

The present study had two main aims. First, we adopted Scientometrics to identify the intellectual structure of language assessment research published in English peer-reviewed journals. Although Scientometrics and similar approaches such as Bibliometric have been adopted in applied linguistics to investigate the knowledge structure across several research domains (Arik and Arik, 2017; Lei and Liu, 2019), there is currently no study that has investigated the intellectual structure of research in language assessment. Here, intellectual structure refers to a set of research clusters that represents specialized knowledge groups and research themes, as well as the growth of the research field over time (Goswami and Agrawal, 2019). To identify an intellectual structure, a representative dataset of the published literature is firstly generated and specialized software is subsequently applied to mine and extract the hidden structures in the data (Chen, 2016). The measures generated are then used to portray the structure and dynamics of the field “objectively,” where the dataset represents the research field in question (Goswami and Agrawal, 2019). Second, we aim to examine the content of emerged research clusters, using two field-specific frameworks to determine how each cluster can be mapped onto commonly adopted methodologies in the field: validity argument (Chapelle, 1998; Bachman, 2005; Kane, 2006; Chapelle et al., 2008; Bachman and Palmer, 2010) and measurement frameworks (Norris and Ortega, 2003). The two research aims are discussed in detail next.

#### First Aim

To achieve the first aim of the study, we adopted a Scientometric technique known as document co-citation analysis (DCA) (Chen, 2006, 2010) to investigate the intellectual structure for the field of language assessment as well as assessment-based research in second language acquisition (SLA). Co-citation refers to the frequency with which two or more publications are referenced in another publication (Chen, 2003, 2016). When a group of publications cites the same papers and books, this means that they are not only thematically related but they also take reference from the same pool of papers (Chen, 2003). Moreover, co-citations can be also generalized to authors and journals by identifying the frequency with which they have been written by the same authors or cited using the same journal resource (Chen, 2004, 2006; Chen and Song, 2017). Of note, co-citation analysis is similar to factor analysis that is extensively used for data reduction and pattern recognition in surveys and tests. In the latter, items are categorized into separate clusters called factors based on their correlation patterns. Factor loadings indicate the correlation of the item in question with other items that are categorized as a factor (Field, 2018). Some items have high loadings on latent variables, whereas others have low loading coefficients. The items with low loading coefficients do not make a significant contribution to the measurement of the ability or skill under assessment and can be removed from the instrument without affecting the amount of variance explained by the test items (Field, 2018). Similarly, co-citation analysis categorizes publications as discrete research clusters based on the publications that are co-cited in each cluster. When two publications co-cite a source or reference, this suggests that they may be related. If these publications share (co-cite) at least 50% of their references, it is plausible that there is a significant thematic link between them. Identifying the publications that co-cite the same sources facilitates the identification of the related research clusters via their pool of references. The publications that are clustered together (like factors in factor analysis) may be then inspected for their thematic relationships, either automatically through text-mining methods or manually by experts who read the content of the clustered publications. Furthermore, there may be influential publications in each cluster that have received large numbers of co-citations from other publications, and this is termed as “citation bursts.” Reviewing the content of the citation bursts can further help researchers characterize the cluster in terms of its focus and scope (Chen, 2017).

#### Second Aim

To achieve the second aim of the study, we developed a framework to describe measurement and validation practices across the emerged clusters. Despite the assumption that testing and assessment practices are specific to the language assessment field, SLA researchers have employed certain assessment techniques to investigate research questions pertinent to SLA (Norris and Ortega, 2003). Nevertheless, there seems to be methodological and conceptual gaps in assessment between the language testing field and SLA, which several publications attempted to bridge (Upshur, 1971; Bachman, 1990; see chapters in Bachman and Cohen, 1998). Bachman (1990, p. 2) asserted that “language testing both serves and is served by research in language acquisition and language teaching. Language tests, for example, are frequently used as criterion measures of language abilities in second language acquisition research.” He extended the uses and contributions of language assessment to teaching and learning practices, stressing that language tests are used for a variety of purposes like assessing progress and achievement, diagnosing learners' strengths and weaknesses, and as tools for SLA research. He stressed that insights from SLA can reciprocally assist language assessment experts to develop more useful assessments. For example, insights from SLA research on learners' characteristics and personality can help language testing experts to develop measurement instruments to investigate the effect of learner characteristics on assessment performance. Therefore, in Bachman's (1990) view, the relationship between SLA and language assessment is not exclusively unidirectional or exclusive to validity and reliability matters. Despite this, doubts have been voiced regarding the measurement of constructs in SLA (Bachman and Cohen, 1998) and the validity of the instruments used in SLA (Chapelle, 1998). For example, Norris and Ortega (2003) critiqued SLA research on the grounds that measurement is not often conducted with sufficient rigor.

Measurement is defined as the process of (i) construct representation, (ii) construct operationalization, (iii) data collection via “behavior elicitation” (Norris and Ortega, 2003, p. 720), (iv) data analysis to generate evidence, and (v) the employment of that evidence to draw theory-based conclusions (Messick, 1989, 1996). To establish whether measurement instruments function properly, it is essential to investigate their reliability and, where applicable and plausible, validate interpretations and uses of their results (scores) (Messick, 1996; Kane, 2006). Reliability refers to the evidence that the measurement is precise or has low error of measurement (Field, 2018) and its output is reproducible across occasions, raters, and test forms (Green and Salkind, 2014; Grabowski and Oh, 2018). In addition, since the publication of Cronbach and Meehl's (1955) paper, validation has been primarily treated as the process of developing arguments to justify the meaning and utility of test scores or assessment results. Messick (1989) emphasized that validation should encompass evidentiary and consequential bases of score interpretation and meaning and Kane (2006) proposed a progressive plan for collecting various sorts of evidence to buttress inferences drawn from the data and rebut counter-evidence (if any). Like the theory of measurement, Messick's (1989) and Kane's (2006) frameworks have had a lasting impact on language assessment (Bachman, 2005; Chapelle et al., 2008; Bachman and Palmer, 2010; Aryadoust, 2013).

We note that, in addition to the argument-based validation framework, there are several validation frameworks such as Weir's (2005b) socio-cognitive framework or Borsboom and Mellenbergh's (2007) test validity framework which have been adopted in some previous research. However, Borsboom and Mellenbergh's (2007) work is less well-known in language assessment and SLA and has a heavy focus on psychometrics. In addition, certain components of Weir's (2005a) framework such as cognitive validity are relatively under-researched in language assessment and SLA and coding the studies for these components would not generate as useful information. Therefore, the choice of argument-based validation framework seems to be more plausible for this study, although we do recognize the limitations of the approach (see *Conclusion*).

Bachman (2005) stressed that, before using an assessment for decision-making purposes, a validity argument should be fully fledged in terms of evidence supporting test developers' claims. On the other hand, empirical validation studies have demonstrated that collecting such evidence to establish an all-encompassing validity argument is an arduous and logistically complex task (Chapelle et al., 2008; Aryadoust, 2013; Fan and Yan, 2020). We are, hence, keen to determine the extent to which language assessment and SLA studies involving measurement and assessment have fulfilled the requirements of validation in the research clusters that are identified through DCA.

## Methodology

### Overview

This study investigated the intellectual structure in the language assessment field. It examines the literature over the period 1918–2019 to identify the network structure of influential research domains involved in the evolution of language assessment. The year 1918 is the lower limit as it is the earliest year of coverage by Scopus. The study adopted a co-citation method that comprises document co-citation analysis (DCA) (Small and Sweeney, 1985; Chen, 2004, 2006, 2010, 2016; Chen et al., 2008, 2010). The study also adopted CiteSpace Version 5.6.R3 (Chen, 2016), a computational tool used to identify highly cited publications and authors that acted as pivotal points of transition within and among research clusters (Chen, 2004).

### Data Source and Descriptive Statistics

Scopus was employed as our main database, with selective searches carried out to create the datasets of the study. We identified several publications that defined language assessment as the practice of assessing first, second or other languages (Hornberger and Shohamy, 2008), including the assessment of what is known to be language “skills and elements” or a combination of them. Despite the defined scope, the bulk of the publications concerns SLA (as will be seen later). We treated the journals that proclaimed their focus to be exclusively language assessment as the “core journals” of the field, while using a keyword search to identify the focus of language assessment publications in applied linguistics/SLA journals. Accordingly, two datasets were created (see Appendix for the search code).

- A core journals dataset consisting of 1,561 articles published in *Language Testing, Assessing Writing, Language Assessment Quarterly*, and *Language Testing in Asia*, which were indexed in Scopus. These journals focus specifically on publishing language assessment research and were, accordingly, labeled as core journals. The dataset also included all the publications (books, papers etc.) that were cited in the *References* of these articles.
- A general journals dataset consisting of 3,175 articles on language assessment published in the top 20 journals of applied linguistics/SLA. The dataset also included all the publications cited in these articles. This list of journals was identified based on their ranking in the “Scimago Journal and Country Rank (SJR)” database and their relevance to the current study. The journals consisted of *Applied Psycholinguistics, System, Language Learning, Modern Language Journal, TESOL Quarterly, Studies in Second Language Acquisition, English Language Teaching, RELC Journal, Applied Linguistics, Journal of Second Language Writing, English for Specific Purposes, Language Awareness, Language Learning and Technology, Recall, Annual Review of Applied Linguistics*, and *Applied Linguistics Review*. There was no overlap between *i* and *ii*. To create *ii*, the Scopus search engine was set to search for generic keywords consisting of “test,” “assess,” “evaluate,” “rate,” and “measure” in the titles, keywords, or abstracts of publication^2^. These search words were chosen from the list of high-frequency words that were extracted by Scopus from the core journal dataset (*i*). Next, we reviewed the coverage of 1,405 out of 3,175 articles^3^, as determined by CiteSpace analysis, that contributed to the networks in this dataset to ascertain if they addressed a topic in language assessment. The publications were found to either have an exclusive focus on assessment or used assessment methods (e.g., test development, reliability analysis, or validation) as one of the components in the study.

Supplemental Table 1 presents the total number of articles published by the top 20 journals, countries/regions, and academic institutes. The top three journal publishers were *Language Testing, System*, and *Language Learning*, with a total of 690, 389, and 361 papers published between 1980 and 2019—note that there were language testing/assessment studies published earlier in other journals. In general, the journals published more than 100 papers, with the exceptions of *Language Learning Journal, ReCall, Language Awareness, Journal of Second Language Writing, Language Learning and Technology*, and *English for Specific Purposes*. The total number of papers published by the top five journals (2,087) accounted for more than 50% of the papers published by all journals.

The top five countries/regions producing the greatest number of articles were *the United States (US), the United Kingdom, Canada, Iran*, and *Japan*, with 1,644, 448, 334, 241, and 233 articles, respectively. Eleven of the top 20 countries/regions, listed in Supplemental Table 1, published more than 100 articles. The top three academic institutes publishing articles were *the Educational Testing Service* (*n* = 99), *the University of Melbourne* (*n* = 92), and *Michigan State University* (*n* = 68). In line with the top producing country, just over half of these institutions were located in the US.

### First Aim: Document Co-Citation Analysis (DCA)

The document co-citation (DCA) technique was used to measure the frequency of earlier literature co-cited together in later literature. DCA was used to establish the strength of the relationship between the co-cited articles, identify ‘popular’ publications with high citations (bursts) in language assessment, and identify research clusters comprising publications related via co-citations^4^. DCA was conducted twice—once for each dataset obtained from Scopus, as previously discussed. We further investigated the duration of burstness (the period of time in which a publication continued to be influential) and burst strength (the quantified magnitude of influence).

#### Visualization and Automatic Labeling of Clusters

The generation of a timeline view on CiteSpace allowed for clusters of publications to be visualized on discrete horizontal axes. Clusters were arranged in a vertical manner descending in size, with the largest cluster at the top. Colored lines representing co-citation links were added in the time period of the corresponding color. Publications that had a citation burst and/or were highly cited were represented with red tree rings or appear larger than the surrounding nodes.

The identified clusters were automatically labeled. In CiteSpace, three term ranking algorithms can be used to label clusters: latent semantic indexing (LSI), log-likelihood ratio (LLR), or mutual information (MI). The ranking algorithms use different methods to identify the cluster themes. LSI uses document matrices but is “underdeveloped” (Chen, 2014, p.79). Both LLR and MI identify cluster themes by indexing noun phrases in the abstracts of citing articles (Chen et al., 2010), with different ways of computing the relative importance of said noun phrases. We chose the labels selected by LLR (rather than MI) as they represent unique aspects of the cluster (Chen et al., 2010) and are more precise at identifying cluster themes (Aryadoust and Ang, 2019).

While separate clusters represent discrete research themes, some clusters may consist of sub-themes. For example, our previous research indicated that certain clusters are characterized by publications that present general guidelines on the application of quantitative methods alongside publications focused on a special topic, e.g., language-related topics (Aryadoust and Ang, 2019; Aryadoust, 2020). In such cases, subthemes and their relationships should be identified (Aryadoust, 2020).

#### Temporal and Structural Measures of the Networks

To evaluate the quality of the DCA network, temporal and structural measures of networks were computed. Temporal measures were computed using citation burstness and sigma (∑). Citation burstness shows how favorably an article was regarded in the scientific community. If a publication receives no sudden increase of citations, its burstness tends to be close or equal to zero. On the other hand, there is no upper boundary for burstness. The sigma value of a node in CiteSpace merges the citation burstness and betweenness centrality, demonstrating both the temporal and structural significance of a citation. Sigma could also be indicative of novelty, detecting publications that presented novel ideas in their respective field (Chen et al., 2010). That is, the higher the sigma value, the higher the likelihood that the publication includes novel ideas.

Structural measures comprised the average silhouette score, betweenness centrality, and the modularity (*Q*) index. The average silhouette score ranges between −1 and 1 and measures the quality of the clustering configuration (Chen, 2019). This score defines how well a cited reference matches with the cluster in which it has been placed (vs. other clusters), depending on its connections with neighboring nodes (Rousseeuw, 1987). A high mean silhouette score suggests a large number of citers leading to the formation of a cluster, and is therefore reflective of high reliability of clustering; by contrast, a low silhouette score illustrates low homogeneity of clusters (Chen, 2019).

The modularity (*Q*) index ranges between −1 and 1 and determines the overall intelligibility of a network by decomposing it into several components (Chen et al., 2010; Chen, 2019). A low *Q* score hints at a network cluster without clear boundaries, while a high *Q* score is telling of a well-structured network (Newman, 2006).

The betweenness centrality metric ranges between 0 and 1 and assesses the degree to which a node is in the middle of a link that connects to other nodes within the network (Brandes, 2001). Moreover, a high betweenness centrality indicates that a publication may contain groundbreaking ideas; if a node is the only connection between two large but otherwise unrelated clusters, this is evidence that the author scores are high on betweenness centrality (Chen et al., 2010).

However, it must be noted that these measures are not absolute scales where a higher value automatically indicates increased importance. Rather, they show tendencies and directions for the analyst to pursue. In practice, one should also consider the diversity of the citing articles (Chen et al., 2010). For example, a higher silhouette value generated from a single citing article is not necessarily indicative of greater importance than a relatively lower value from multiple distinct citing articles. Likewise, the significance of the modularity index and the betweenness centrality metric is subject to interpretation, dependent on further analyses, including of citing articles.

### Second Aim: The Analytical Framework

In DCA, clusters reflect what *citing* papers have in common in terms of how they cite references together (Chen, 2006). Therefore, we designed an analytical framework to examine the citing publications in the clusters (Table 1). In addition, we took into account the bursts (cited publications) per cluster in deciding what features would characterize each cluster. The framework was informed by a number of publications in language assessment research such as Aryadoust (2013), Bachman (1990), Bachman and Cohen (1998), Bachman and Palmer (2010), Chapelle et al. (2008), Eckes (2011), Messick (1989), Messick (1996), Kane (2006), Norris and Ortega (2003), and Xi (2010a). In Table 1, “component” is a generic term to refer to the inferences that are drawn from the data and are supported by warrants (specific evidence that buttress the claims or conclusions of the data analysis) (Kane, 2006; Chapelle et al., 2008; Bachman and Palmer, 2010). In addition, it also refers to the facets of measurement articulated by Messick (1989, 1996) and Norris and Ortega (2003) in their investigation of measurement and construct definition in assessment and SLA. It should be noted that the validity components in this framework, i.e., generalization, explanation, extrapolation, and utilization, are descriptive (rather than evaluative) and intended to record whether or not particular studies reported evidence for them. Thus, the lack of reporting of these components does not necessarily indicate that this evidence was not presented when it should have been, unless it is stated otherwise.

**Table 1:** The analytical framework to address the second aim of the study.

**Component**	**Definition**	**Relevant procedures and/or warrants**	**References**
Domain specification	The definition of the target language use (TLU) domain and the components of the representation of the construct in question (construct representation)	Generating a theoretical framework to explain (i) the cognitive processes of the latent trait under investigation (competency-based approach) and/or (ii) the characteristics of the tasks that represent the TLU domain (task-based approach)	Messick, 1989; Norris and Ortega, 2003; Chapelle et al., 2008
Construct operationalization	The realization of the construct or translating the construct definition into actual assessment instruments	(i) Using one or more task formats such as open-ended questions or discrete-point/selected response methods like multiple choice questions, and (ii) experts' evaluation of the tasks	Messick, 1989; Norris and Ortega, 2003
Evaluation (scoring)	Eliciting the intended behavior from the test taker and using a scale to translate the test performance to a score, mark, or grade	(i) Developing or adapting a scale to grade or provide feedback on students' performance. This can be conducted by human raters or machines (e.g., automated writing evaluators), (ii) establishing the reliability of the scale using reliability analysis (e.g., internal consistency or rater reliability)	Norris and Ortega, 2003; Kane, 2006; Chapelle et al., 2008; Bachman and Palmer, 2010; Xi, 2010a; Grabowski and Oh, 2018
Generalization	Establishing whether the observed scores represent a “universe score” and are not exclusive to the test form, rater, or test item formats in the assessment	Generalizability theory analysis or many-facet Rasch measurement to investigate the sources of variance and error in data as well as the erratic marking patterns.	Kane, 2006; Eckes, 2011; Aryadoust, 2013; Grabowski and Lin, 2019; Sawaki and Xi, 2019
Explanation (analogous to traditional construct validation)	Establishing whether the test engages the target construct or whether the test takers' performance can primarily be explained by the target construct	Latent variable analysis such as exploratory or confirmatory factor analysis or Rasch measurement	Chapelle et al., 2008
Extrapolation (analogous to traditional criterion evidence of validity)	Establishing whether the test scores can be extrapolated to or predict test takers' performance in the TLU domain	Correlation analysis, regression analysis, or structural equation modeling (SEM) to examine the relationships between test results and future performance of the test takers in the TLU domain	Kane, 2006; Bachman and Palmer, 2010
Utilization (analogous to traditional washback research or consequential validity)	Establishing whether the test results are used appropriately and whether their use has any positive impact on the individual, educational system, and society	Investigation of washback through collecting evidence from classrooms, work places, or test takers, using questionnaires or interviews and analysis methods such as SEM or regression analysis.	Bailey, 1999; Bachman and Palmer, 2010;

Using this framework, we coded the publications independently and compared their codes. Only few discrepancies were identified which were subsequently resolved by the first author.

## Results

### DCA of the Core and General Journals Networks

Supplemental Table 2 presents the top publications in the core and general journals datasets with the strongest citation bursts sustained for at least 2 years. (Due to space constraints, only the top few publications have been presented). Overall, the publications had a low betweenness centrality index ranging from 0.01 to 0.39. Bachman (1990; centrality = 0.35) and Canale and Swain (1980; centrality = 0.39) had the highest betweenness centrality index among the core and general journals datasets, respectively. Of these, Bachman (1990) and Skehan (1998) appeared on both core and general journals lists. The books identified in the analysis were not included directly in the datasets; they appeared in the results since they were co-cited by a significant number of citing papers (i.e., they came from the *References* section of the citing papers).

The top five most influential publications in the core journals were Bachman and Palmer (1996; duration of burst = 6, strength = 17.39, centrality = 0.11, sigma = 6.4), Bachman and Palmer (2010; duration of burst = 4, strength = 14.93, centrality = 0.02, sigma = 1.25), Bachman (1990; duration of burst = 5, strength = 11.77, centrality = 0.35, sigma = 32.79), Fulcher (2003; duration of burst = 5, strength = 11.54, centrality = 0.01, sigma = 1.10), and Council of Europe (2001; duration of burst = 3, strength = 11.17, centrality = 0.01, sigma = 1.11).

In addition, four publications in the general journals dataset had a burst strength higher than 11: Skehan (1988; duration of burst = 9, strength = 13.42, centrality = 0.05, sigma = 1.85), Bachman and Palmer (1996; duration of burst = 7, strength = 12.15, centrality = 0.05, sigma = 1.81), Norris and Ortega (2009; duration of burst = 7, strength = 13.75, centrality = 0.01, sigma = 1.08), and Nation (1990; duration of burst = 6, strength = 11.00, centrality = 0.05, sigma = 1.67).

### Visualization of the DCA Network for the Core Journals Dataset

**Figure 1:**
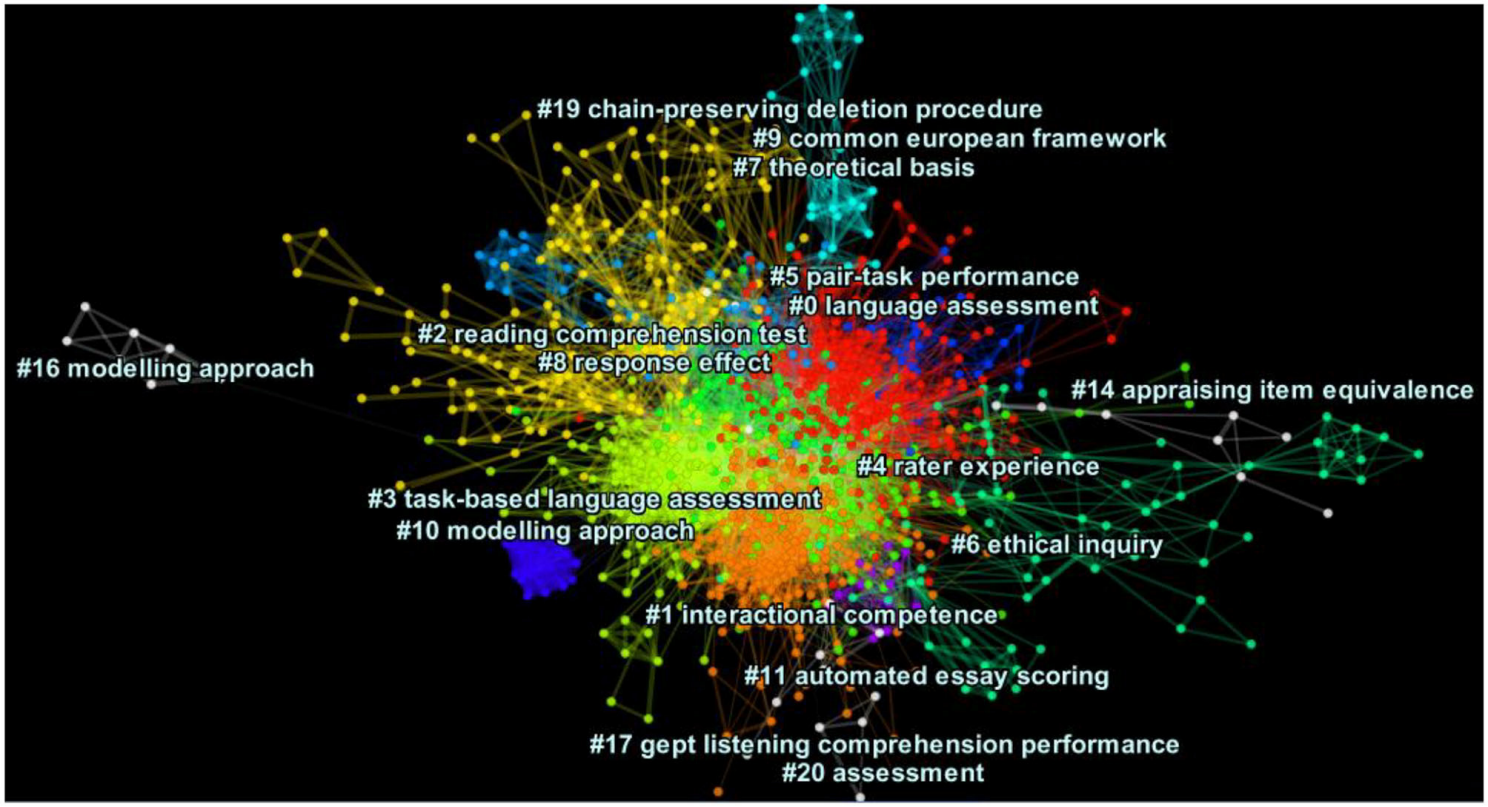
The cluster view of network in the core journals dataset (modularity Q = 0.541, average silhouette score = 0.71), generated using CiteSpace, Version 5.6.R3.

Figure 1 depicts the cluster view of the DCA network of the core journals. Each cluster consists of nodes, which represent publications, and their links which are represented by lines and show co-citation connections. The labels per clusters are representative of the headings assigned to the citing articles within the cluster. The color of a link denotes the earliest time slice in which the connection was made, with warm colors like red representing the most recent burst and cold colors like blue representing older clusters. As we can see from the denseness of the nodes in Figure 1, there were six largest clusters experiencing citation bursts: #0 or language assessment (size=224; silhouette value = 0.538; Mean year of publication = 1995), #1 or interactional competence (size= 221; silhouette value = 0.544; Mean year of publication = 2005), #2 or reading comprehension test (size= 171; silhouette value =0.838; Mean year of publication = 1981), #3 or task-based language assessment (size= 161; silhouette value = 0.753; Mean year of publication = 1994), # 4 or rater experience (size=108; silhouette value =0.752; Mean year of publication = 1999), and #5 or pair task performance (size = 78; silhouette value = 0.839; Mean year of publication = 1993). Note that the numbers assigned to the clusters in this figure (from 0 to 20) are based on the cluster size, so #0 is the largest, followed by #1, etc. Smaller clusters with too few connections are not presented in cluster views. This DCA network had a modularity Q metric of 0.541, indicating a fairly well-structured network. The average silhouette index was 0.71, suggesting medium homogeneity of the structures (See Supplemental Table 3 for further information). It should be noted that after examining the content of each cluster, we made some revisions to the automatically generated labels to enhance their consistency and precision (see *Discussion*).

### Visualization of the DCA Network for the General Journals

**Figure 2:**
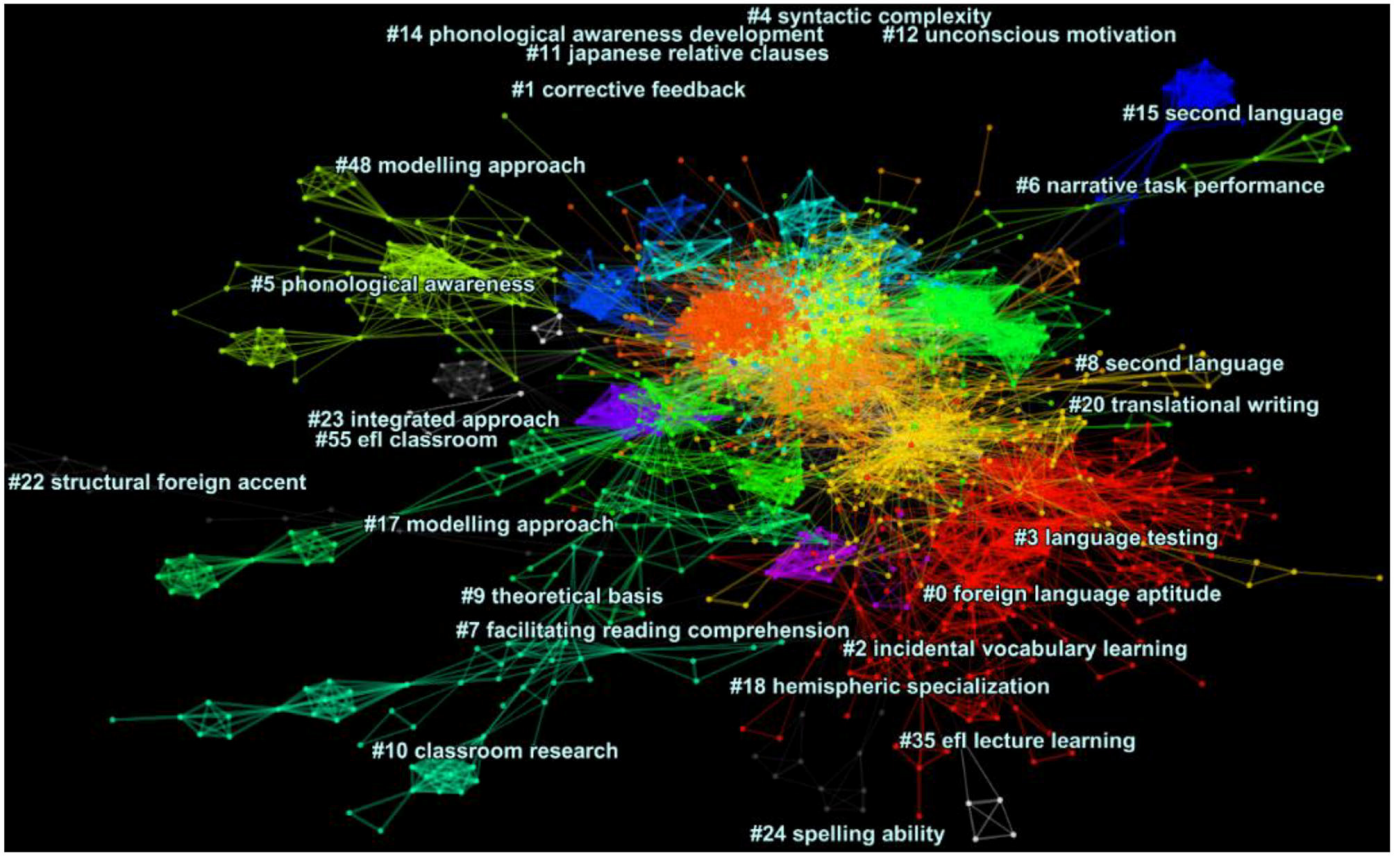
The cluster view of network in the general journals dataset (modularity Q = 0.6493, average silhouette score = 0.787), generated using CiteSpace, Version 5.6.R3.

Figure 2 depicts a cluster view of the major clusters in the general journals dataset visualized along multiple horizontal lines (modularity Q = 0.6493, average silhouette score = 0.787). The clusters are color-coded, with their nodes (publications) and links being represented by dots and straight lines, respectively. Among the clusters visually represented, there were nine major clusters in the network, as presented in Supplemental Table 4. The largest cluster is #2 (incidental vocabulary learning); the oldest cluster is #0 (foreign language aptitude), whereas the most recent one is #4 (syntactic complexity). As presented in the Supplemental Table 4, although the dataset represented co-citation patterns in the general journals, we noted that there were multiple cited publications in this dataset that were published in the core journals. It should be noted that only major clusters are labeled and displayed in Figures 1, 2 and therefore the running order of the clusters are different across the two.

### Second Aim: Measurement and Validity in the Core Journal Clusters

Next, we applied the analytical framework of the study in Table 1 to examine the measurement and validation practices in each main cluster.

#### Domain Specification in Core Journals

For the core dataset, Table 2 presents the domains and constructs specified in the six major clusters. (Please note that the labels under the “The construct or domain specified” column were inductively assigned by the authors based on the examination of papers in each cluster). Overall, there were fewer constructs/domains in the core dataset (*n* = 15) as compared to the 26 in the general journals dataset below. The top four most frequently occurring constructs or domains in the core dataset were speaking/oral/communicative skills, writing and/or essays, reading, and raters/ratings. The most frequently occurring construct, Speaking/oral/communicative skills, appeared in every cluster, which is indicative of one of the major foci of the core journals. A series of χ^2^ tests showed that all categories of constructs or domains were significantly different from each other in terms of the distribution of the skills and elements (*p* < 0.05). Specifically, Clusters #0 and #2 were primarily characterized by the dominance of comprehension (reading and listening) assessment research while Clusters #1, #4, and #5 had a heavier focus on performance assessment (writing and oral production/interactional competence), thus suggesting two possible streams of research weaving the clusters together. The assessment of language elements such as vocabulary and grammar was significantly less researched across all the clusters.

**Table 2:** Domain specification in major clusters in the core journals.

**Cluster #**	**The construct or domain specified**	**# of papers**
**Cluster 0**		
	Reading	18
	Listening	8
	Speaking/ oral/ communicative ability	8
	Writing	5
	Overall language proficiency	7
**Cluster 1**		
	Reading	8
	Writing	29
	Speaking/ oral/ communicative ability	16
	Interactional competence	6
	Corpus linguistics	3
	Overall language proficiency	9
	Feedback	3
**Cluster 2**		
	Reading	6
	Listening	2
	Speaking/ oral/ communicative ability	3
**Cluster 3**		
	Reading	3
	Vocabulary	7
	Speaking/ oral/ communicative	5
	Overall language proficiency	2
**Cluster 4**		
	Vocabulary	3
	Writing/ essays	15
	Raters/ ratings	18
	Speaking/ oral/ communicative ability	8
**Cluster 5**		
	Speaking/ oral/ communicative ability	13
	Washback	2

#### Other Components in Core Journals

Table 3 presents the other components of the analytical framework in the core journals consisting of construct operationalization, evaluation, generalization, explanation, extrapolation, and utilization. The domains and constructs were operationalized using (i) a discrete-point and selected response format comprising 61 assessments that used cloze, Likert scales, and multiple-choice items, and (ii) production response format comprising 61 essays and writing assessments, and 59 oral production and interview. Specifically, the two most frequently occurring methods of construct operationalization were through cloze/ Likert/ multiple choice and essays and writing assessments in the major clusters of the core journals dataset.

**Table 3:** Measurement methods and evidence of validity in major clusters in the core journals.

**Construct operationalization**				
Cluster ID	Cloze/ Likert/ multiple choice	Essays and writing	Oral/interview	Total
1	10	32	21	63
4	17	17	9	43
0	20	5	13	38
5	4	0	11	15
2	8	4	2	14
3	2	3	3	8
Total	61	61	59	181
**Reliability**				
Cluster ID	Reported reliability	Did not report reliability		Total
1	49	36		85
0	30	29		59
4	26	4		30
3	8	18		26
2	13	8		21
5	10	9		19
**Generalization**				
Cluster ID	Reported generalizability evidence	Did not report generalizability evidence		Total
1	6	79		85
0	1	58		59
4	6	24		30
3	0	26		26
2	1	20		21
5	3	16		19
**Criterion Evidence of Validity**				
Cluster ID	Yes	No		Total
1	5	80		85
0	5	54		59
4	1	29		30
3	2	24		26
2	5	16		21
5	0	19		19
**Utilization**				
Cluster ID	Yes	No		Total
1	1	82		85
0	6	50		59
4	0	27		30
3	0	24		26
2	1	20		21
5	4	14		19
**Explanation**				
Cluster ID	Yes	No		Total
1	10	75		85
0	8	51		59
4	3	27		30
3	0	26		26
2	3	18		21
5	2	17		19

In addition, reliability coefficients were reported in slightly more than half of the publications (56.7%), whereas generalizability was underreported in all the clusters with a mere 7.1% of the studies presenting evidence of generalizability. Likewise, only 7.5% presented criterion-based evidence of validity; 10.8% of the studies reported or investigated evidence supporting construct validity or the explanation inference; and 5% (12/240) of the studies addressed the utilization inference of the language assessments investigated. Among the clusters, Cluster #5 and #0 had the highest respective ratios of 4/19 (21%) and 6/59 (10%) studies investigating the utilization inference.

### Measurement and Validity in the General Journal Clusters

#### Domain Specification in General Journals

Table 4 presents the domains and constructs specified in the major clusters in the general journals dataset. Of the 26 constructs/domains specified in the nine clusters, the top five constructs/domains in the clusters were grammar, speaking/ oral interactions, reading, vocabulary, and writing (ranked by frequency of occurrence in the clusters). Grammar appeared in every cluster except Cluster 8 which was distinct from other clusters as papers in this cluster did not examine linguistic constructs but the affective aspects of language learning, with a relatively low number of publications (*n* = 13). Looking at the number of papers for each respective domain in each cluster, we can observe that some clusters were characterized by certain domains. By frequency of occurrence, papers in Cluster 0 was mostly concerned with language comprehension (reading and listening), whereas Cluster 1 was characterized by feedback on written and oral production; Cluster 2 by vocabulary; and Cluster 4 by writing, with syntactic complexity being secondary in importance. A series of χ^2^ tests showed that 20 of the 26 categories of construct or domains occurred with significantly unequal probabilities, i.e., fluency, speaking, oral ability/proficiency, language proficiency/competence, feedback, collocations, semantic awareness, syntactic complexity, task complexity, phonological awareness, explicit/ implicit knowledge, comprehension, anxiety, attitudes, motivation, relative clauses, and language awareness (*p* < 0.005).

**Table 4:** Domain specification in major clusters in the general journals.

**Cluster #**	**The construct or domain specified**	**# of papers**
**Cluster 0**		
	Reading	12
	Listening	10
	Speaking	6
	Writing	4
	Grammar	5
	Vocabulary	5
	Oral ability	1
	Oral proficiency	1
	Language proficiency	3
	Language competence	1
**Cluster 1**		
	Reading	1
	Listening	1
	Speaking/ Oral/ Interaction	15
	Writing	3
	Grammar	6
	Vocabulary	1
	Memory	4
	Feedback^*^	15
**Cluster 2**		
	Reading	9
	Listening	9
	Speaking/ Oral/ Interaction	1
	Writing	5
	Grammar	1
	Vocabulary	43
	Collocations	5
	Semantic awareness	2
**Cluster 3**		
	Reading	2
	Listening	1
	Speaking/ Oral/ Interaction	5
	Writing	3
	Grammar	2
	Vocabulary	3
**Cluster 4**		
	Speaking/ Oral/ Interaction	5
	Writing	21
	Grammar	3
	Vocabulary	1
	Fluency	5
	Syntactic complexity	7
	Task complexity	2
**Cluster 5**		
	Reading	2
	Speaking/ Oral/ Interaction	2
	Grammar	1
	Vocabulary	3
	Phonological awareness	3
**Cluster 6**		
	Reading	1
	Speaking/ Oral/ Interaction	1
	Grammar	1
	Fluency	2
	Explicit/ implicit knowledge	3
	Listening comprehension	2
**Cluster 8**		
	Anxiety	4
	Attitudes	3
	Motivation	6
**Cluster 11**		
	Grammar	2
	Relative clauses	3
	Language awareness	2

* *Papers on feedback were double-counted in other categories. This consisted of 10 papers on speaking/oral/interaction, 1 paper on grammar, 1 on explicit feedback, 1 on the use of classifiers and the perfective -le in Chinese, and 2 papers on writing*.

#### Other Components in General Journals

Table 5 presents the breakdown of construct operationalization and the presentation of evidence of validity in the papers in the major clusters of the general journals data set. Given the domain characteristics (writing) of Cluster 4, discussed above, it is not surprising that the constructs are operationalized mainly through writing/essay in 59.6% of the papers in the cluster. As with the core journals dataset, the evaluation of reliability in the papers is fairly split, with 54.63% of the publications reporting reliability. The vast majority of papers did not provide any generalizability evidence (98.83%). Likewise, the majority of papers did not investigate construct validity (extrapolation) (95.03%) nor did they provide criterion evidence of validity (93.27%). Finally, only 24 of the publications reported or investigated the utilization inference.

**Table 5:** Measurement practices and evidence of validity in major clusters in the general journals.

**Cluster ID**	**Cloze/Likert/** **multiple choice**	**Essay/writing**	**Oral/interview**	**Total**
**Construct operationalization**				
2	29	13	6	48
1	3	16	21	40
3	10	7	12	29
0	20	8	8	36
4	3	28	16	47
6	6	2	6	14
8	5	0	1	6
5	2	0	6	8
11	3	4	4	11
**Cluster ID**	**Reported reliability**	**Did not report reliability**	**Non-English**	**Total**
**Reliability**				
2	44	40	0	84
1	34	32	0	66
3	21	20	0	41
0	25	13	0	38
4	27	22	0	49
6	16	8	0	24
8	5	6	1	12
5	12	3	0	15
11	3	9	1	13
**Cluster ID**	**Reported generalizability evidence**	**Did not report generalizability evidence**	**Non-English**	**Total**
**Generalization**				
2	1	83	0	84
1	0	66	0	66
3	1	40	0	41
0	0	38	0	38
4	0	49	0	49
6	0	24	0	24
8	0	11	1	12
5	0	15	0	15
11	0	12	1	13
**Cluster ID**	**Yes**	**No**	**non-English**	**Total**
**Criterion Evidence of Validity**				
2	3	81	0	84
1	4	62	0	66
3	5	36	0	41
0	6	32	0	38
4	1	48	0	49
6	0	24	0	24
8	0	11	1	12
5	2	13	0	15
11	0	12	1	13
**Cluster ID**	**Yes**	**No**	**Non-English**	**Total**
**Explanation**				
2	2	82	0	84
1	4	62	0	66
3	4	37	0	41
0	6	32	0	38
4	1	48	0	49
6	0	24	0	24
8	0	12	0	12
5	0	15	0	15
11	0	13	0	13
**Cluster ID**	**Yes**	**No**	**Claimed without evidence**	**Total**
**Utilization**				
2	0	82	2	84
1	0	63	3	66
3	0	29	12	41
0	1	30	7	38
4	0	49	0	49
6	0	24	0	24
8	0	11	0	12
5	0	15	0	15
11	0	12	0	13

## Discussion

This study set out to investigate intellectual domains as well as the use of measurement and validation methods in language assessment research. We created two datasets covering the core and general journals, and employed DCA to detect research clusters. Next, we coded citing papers in each cluster based on an analytical framework for measurement and validation (Norris and Ortega, 2003; Kane, 2006; Bachman and Palmer, 2010). In this section, we will discuss bursts and citing publications per cluster to determine the features that possibly characterize each main clusters. Next, we will discuss the measurement and validation practices in the citing papers in the two datasets.

### First Aim: Characterizing the Detected Clusters

#### Core Journals

Bursts (impactful cited publications) in the influential clusters in the core journals dataset are presented in Table 6. The review presented in the following sections is organized according to the content and relevance of these publications. We will further provide a broad overview of these publications. It should be noted that while narrative literature reviews customarily have specific foci, what we aim to do is to leverage the potentiality of clustering and highlight the linked concepts that might have resulted in the emergence of each cluster. Each cluster will be characterized by virtue of the content of the citing and cited publications. Due to space constraints, we provide a detailed review commentary on two of the largest clusters in the Core Journals dataset, and a general overview of the rest of the major clusters (see the Appendices for further information per cluster).

**Table 6:** Selected cited publications (Bursts) in the core journals.

**References**	**Burst strength**	**Frequency**	**Centrality**	**Sigma**	**Cluster ID**
Bachman and Palmer (1996)	17.39	63	0.11	6.4	0
Alderson et al. (1995)	10.65	28	0.02	1.19	0
Bachman (1990)	9.58	67	0.16	4.13	0
Alderson (2000)	8.55	26	0.01	1.07	0
Bachman and Palmer (2010)	7.97	18	0.01	1.06	0
Shohamy (2001)	7.84	22	0.01	1.1	0
Alderson (2005)	7.7	22	0.02	1.13	0
McNamara (1996)	7.22	22	0.02	1.14	0
Buck (2001)	6.86	18	0	1.02	0
Bond and Fox (2007)	6.55	12	0	1.02	0
Bachman (2005)	5.99	32	0.03	1.17	0
Read (2000)	5.64	13	0	1.01	0
Taylor (2009)	5.33	10	0	1.02	0
Alderson and Hamp-Lyons (1996)	4.7	12	0.01	1.05	0
Douglas (2000)	4.47	8	0	1.01	0
Fulcher (2004)	4.16	11	0.01	1.03	0
Canale and Swain (1980)	4.13	49	0.22	2.29	0
Brennan (2001)	4.06	10	0	1.01	0
Alderson and Lukmani (1989)	3.75	15	0.02	1.07	0
Kobayashi (2002)	3.68	7	0	1.02	0
Davison (2007)	3.64	6	0	1.01	0
Brindley (2001)	3.62	6	0	1.01	0
Fulcher (2003)	11.55	27	0.01	1.1	1
Council of Europe (2001)	11.17	23	0.01	1.11	1
American Educational Research Association (2014)	9.17	19	0.01	1.05	1
Weigle (2002)	9.05	60	0.05	1.6	1
Knoch (2009)	7.77	21	0.01	1.08	1
Kane (2006)	7.3	30	0.03	1.24	1
Weir (2005a)	6.82	16	0.01	1.04	1
Luoma (2004)	6.74	14	0	1.02	1
Guo et al. (2013)	6.29	13	0	1.01	1
Messick (1989)	6.17	81	0.12	2.03	1
Cohen (1988)	5.99	19	0.01	1.07	1
Fulcher et al. (2011)	5.8	10	0	1.02	1
Kane (2013)	5.54	15	0.01	1.04	1
Chapelle et al. (2008)	5.1	12	0	1.02	1
Cumming (2013)	4.81	10	0	1.02	1
Biber and Gray (2013)	4.67	11	0	1.01	1
Iwashita et al. (2008)	4.44	17	0.01	1.05	1
Gebril (2009)	4.33	15	0	1.02	1
Flower and Hayes (1981)	4.32	8	0	1.01	1
McNamara et al. (2014)	4.32	8	0	1.01	1
May (2011)	4.26	10	0	1.01	1
Deane (2013)	4.07	14	0.01	1.03	1
Jacobs (1981)	3.98	7	0	1.02	1
Fulcher (1996)	3.81	15	0.01	1.03	1
Ortega (2003)	3.78	7	0	1	1
Plakans (2008)	3.69	11	0	1.02	1
Knoch (2011)	3.69	10	0.01	1.03	1
Wright and Stone (1979)	8.1	17	0.05	1.48	2
Henning (1987)	6.09	13	0.02	1.14	2
Oller (1979)	5.29	9	0.04	1.25	2
Rasch (1960)	5.25	8	0.01	1.05	2
Hambleton and Swaminathan (1985)	4.91	8	0.01	1.06	2
Hughes (1989)	4.55	7	0.01	1.05	2
McNamara (1990)	4.21	8	0.01	1.03	2
Chen and Henning (1985)	4.02	8	0.03	1.14	2
Skehan (1998)	7.9	16	0.01	1.1	3
Messick (1989)	7.18	12	0.01	1.05	3
Brindley (1998)	5.52	12	0.04	1.22	3
Clapham (1996)	4.8	8	0.01	1.03	3
Messick (1994)	4.58	12	0.03	1.12	3
Brown and Hudson (1998)	3.89	6	0.01	1.02	3
Bachman (1990)	3.73	6	0	1	3
Alderson and Wall (1993)	3.61	19	0.01	1.05	3
Cumming et al. (2002)	8.48	26	0.01	1.1	4
Lumley (2002)	7.94	43	0.04	1.32	4
Cumming (1990)	6.72	28	0.01	1.09	4
Eckes (2008)	6.05	24	0.01	1.06	4
Lumley and McNamara (1995)	5.27	26	0.01	1.07	4
Weigle (1998)	4.54	36	0.03	1.14	4
Weigle (1994)	4.49	17	0.01	1.04	4
Brown (1995)	4.26	22	0.04	1.17	4
Lim (2011)	4.06	7	0	1	4
Barkaoui (2010)	3.83	9	0	1	4
(Hamp-Lyons, 1991)	3.81	13	0.01	1.04	4
Brown (2003)	6.65	28	0.02	1.15	5
van Lier (1989)	4.81	13	0.02	1.08	5
Lazaraton (1996)	4.59	14	0.01	1.05	5
Messick (1996)	4.15	33	0.03	1.14	5
Chalhoub-Deville (2003)	3.95	17	0.01	1.04	5
Shohamy (1988)	3.88	6	0.01	1.03	5

#### Cluster 0: Language assessment (and comprehension)

As demonstrated in Table 7, bursts in this cluster can roughly be divided into two major groups: (i) generic textbooks or publications that present frameworks for the development of language assessments in general (e.g., Bachman, 1990; Alderson et al., 1995; Bachman and Palmer, 1996, 2010; McNamara, 1996; Shohamy, 2001; Alderson, 2005), or of specific aspects in the development of language assessments (Alderson, 2000; Read, 2000; Brennan, 2001; Buck, 2001; Kobayashi, 2002; Bachman, 2005) and psychometric measurement (McNamara, 1996; Bond and Fox, 2007), and (ii) publications that describe the contexts and implementations of tests (Alderson and Hamp-Lyons, 1996; Fulcher, 2004; Davison, 2007; Taylor, 2009). The citing publications in this cluster, on the other hand, consist of papers that chiefly investigate the assessment of comprehension skills (The labels under *Focus area 1* and *Focus area 2* in Tables 7, 8 and Supplemental Tables 5 through 11 were inductively assigned by the authors based on the examination of papers).

**Table 7:** Major citing and cited publications in clusters 0 in the core journals.

**Cluster**	**References**	**Citing**	**Cited (bursts)**	**Focus area 1**	**Focus area 2**
0	(Bachman and Palmer, 1996)		X	Test usefulness	Test development
0	(Alderson et al., 1995)		X	Test specification	Test development
0	(Bachman, 1990)		X	Test development	Test methods facets
0	(Alderson, 2000)		X	Test development (reading)	-
0	(Bachman and Palmer, 2010)		X	Validation	Test development
0	(Shohamy, 2001)		X	Tests and policy-making	Democratic assessment
0	(Alderson, 2005)		X	Test development (diagnostic assessment)	The DIALANG assessment system
0	(McNamara, 1996)		X	Test development	Psychometric measurement
0	(Buck, 2001)		X	Test development (listening)	Theories of listening
0	(Bond and Fox, 2007)		X	Rasch measurement	-
0	Bachman (2005)		X	Validation	-
0	(Read, 2000)		X	Test development (Vocabulary)	Theories of vocabulary acquisition and assessment
0	(Taylor, 2009)		X	Language assessment literacy	Test wiseness
0	(Alderson and Hamp-Lyons, 1996)		X	Washback	The TOEFL
0	(Douglas, 2000)	X	X	Assessment of language for specific purposes	-
0	(Fulcher, 2004)		X	The Common European Framework of Reference	Language assessment (political dimensions)
0	(Canale and Swain, 1980)		X	Communicative competence framework	-
0	Brennan (2001)		X	Generalizability theory	-
0	(Kobayashi, 2002)		X	Test method effect	-
0	(Davison, 2007)		X	Hong Kong Examinations and Assessment Authority (HKEAA) School Based Assessment	Perceptions toward school-based assessments
0	(Harsch, 2014)	X		Review of General Language Proficiency	-
0	(McNamara, 2014)	X		Review of Communicative Language Testing (Editorial)	CEF
0	(Phakiti and Roever, 2011)	X		Review of Language Assessment in Australia and New Zealand (Editorial)	-
0	(Xi, 2010b)	X		Review of Automated scoring and feedback systems (Editorial)	-
0	(Lee and Sawaki, 2009)	X		Review of cognitive diagnostic assessment	-
0	(Carr, 2006)	X		Reading comprehension	Test task characteristics
0	(Zhang et al., 2014)	X		Reading comprehension	-
0	(Papageorgiou et al., 2012)	X		Listening comprehension	Test task characteristics (Dialogic vs. monologic assessment)
0	(Roever, 2006)	X		Pragmalinguistics	Validity
0	(Winke, 2011)	X		U.S. Naturalization Test	Reliability
0	Gao and Rogers (2011)	X		Reading comprehension	Test task characteristics
0	(Green and Weir, 2010)	X		Reading comprehension (textual features)	Validity
0	(Jang, 2009a)	X		Reading comprehension	Cognitive diagnostic assessment
0	(Jang, 2009b)	X		Reading comprehension	Cognitive diagnostic assessment
0	(Sawaki et al., 2009)	X		Reading and listening comprehension	Cognitive diagnostic assessment
0	(Harding et al., 2015)	X		Reading and listening comprehension	Diagnostic assessment
0	(Eckes and Grotjahn, 2006)	X		(German) General Language Proficiency (reading, listening, writing, speaking)	Validity

**Table 8:** Major citing and cited publications in clusters 1 in the core journals.

**Cluster**	**References**	**Citing**	**Cited (bursts)**	**Focus area 1**	**Focus area 2**
1	(Fulcher, 2003)		X	Speaking	
1	(Council of Europe, 2001)		X	Assessment	
1	American Educational Research Association, American Psychological Association, and National Council on Measurement in Education, 2014		X	Assessment	Validation
1	(Weigle, 2002)		X	Writing	
1	(Knoch, 2009)		X	Rating scales	Writing
1	(Kane, 2006)		X	Validation	
1	(Weir, 2005a)		X	Validation	
1	(Luoma, 2004)		X	Speaking assessment	
1	(Guo et al., 2013)		X	Linguistic features and rating	Coh-Metrix
1	(Messick, 1989)		X	Validation	
1	(Fulcher et al., 2011)		X	Rating scales	Speaking
1	(Kane, 2013)		X	Validation	
1	(Chapelle et al., 2008)		X	Validation	
1	(Cumming, 2013)		X	Review of Integrated Writing Tasks	
1	(Iwashita et al., 2008)		X	Rating scales	Speaking
1	(Gebril, 2009)		X	Integrated Writing Tasks	
1	(Flower and Hayes, 1981)		X	Writing process	
1	(McNamara et al., 2014)		X	Coh-Metrix	Linguistic features
1	(May, 2011)		X	Rating scales	Speaking
1	(Deane, 2013)		X	Automated scoring	Writing
1	(Jacobs, 1981)		X		
1	(Fulcher, 1996)		X	Rating scales	Speaking
1	(Ortega, 2003)		X	Review of syntactic complexity	
1	(Plakans, 2008)		X	Integrated Writing Tasks	
1	(Knoch, 2011)		X	Rating scales	Writing
1	(Plakans et al., 2019)	X		Integrated writing tasks (reading-writing)	Process
1	(Plakans and Gebril, 2017)	X		Integrated (reading-listening-writing) tasks	The TOEFL iBT
1	(Banerjee et al., 2015)	X		Writing assessment	Rating scale
1	(Barkaoui and Knouzi, 2018)	X		Writing assessment	Mode effect
1	(Guo et al., 2013)	X	X	Writing assessment	Linguistic features
1	(Isbell, 2017)	X		Writing assessment	Rating
1	(Lallmamode et al., 2016)	X		Writing assessment	Validation of scoring rubric
1	(Lu, 2017)	X		Writing assessment	Syntactic Complexity
1	(Rakedzon and Baram-Tsabari, 2017)	X		Writing assessment	Scoring rubric
1	(Wilson et al., 2017)	X		Writing assessment	Automated scoring (using linguistic features measures)
1	(Zhao, 2017)	X		Writing assessment	Scoring rubric (Voice)
1	(Zheng and Yu, 2019)	X		Writing assessment	Review of writing assessment
1	(Lam, 2018)	X		Speaking assessment	Interactional competence
1	(van Batenburg et al., 2018)	X		Speaking assessment	Interactional competence
1	(Römer, 2017)	X		Speaking assessment	Lexicogrammar

Among the bursts in the first group, a few publications prove to be the pillars of the field: Alderson et al. (1995), Bachman (1990), and Bachman and Palmer (1996, 2010). This can be seen from the burst strength of these publications (Table 6) as well as from the citing publications. The articles that cite the publications in Cluster 0 span from reviews or editorials that provide an overview of the field of language assessment to looking at aspects of language assessment. Reviews of the field of language assessment (e.g., Harsch, 2014; McNamara, 2014) consistently mention the works of Bachman. Bachman's influence is such that his publications merited mention even when reviewing specific areas in the field as in Phakiti and Roever (2011) on regional issues in Australia and New Zealand, Xi (2010b) on scoring and feedback, and Lee and Sawaki (2009) on cognitive diagnostic assessment. Bachman and Palmer (1996, 2010) have wide appeal and are referenced with respect to a wide range of topics like reading (Carr, 2006; Zhang et al., 2014), listening (Papageorgiou et al., 2012), and pragmalinguistics (Roever, 2006) in Cluster 0. Bachman and Palmer (1996) and Bachman (1990) are also frequent sources for definitions, examples of which are too numerous to recount exhaustively. Two examples are that of reliability in Winke (2011) and of practicality in Roever (2006), which show the influence of these two texts in explicating core concepts of language assessment.

Articles on the assessment of reading comprehension (e.g., Jang, 2009a,b; Sawaki et al., 2009; Green and Weir, 2010; Gao and Rogers, 2011; Harding et al., 2015) often reference Charles Alderson: Alderson (2000), Alderson (2005) and to a lesser extent, Alderson et al. (1995) and Alderson and Lukmani (1989). For example, Jang's (2009a,b) studies on reading comprehension investigated the validity of LanguEdge test materials and the notion of reading subskills using cognitive diagnosis assessment. Prior discussions on the various aspects of reading assessment—like subskills—in Alderson's various works feature strongly in such studies (see also Sawaki et al., 2009). An exception is Carr's (2006) study on reading comprehension. While mentioning Alderson (2000), Bachman and Palmer's (1996) task characteristics model undergirds Carr's (2006) investigation on the relationship between test task characteristics and test taker performance.

Just like Alderson's works for reading, Buck (2001) seems to be the definitive textbook on assessing the listening component of language. For example, in influential citing papers such as Harding et al. (2015), Papageorgiou et al. (2012), as well as Sawaki et al. (2009), Buck's conceptualization of the subskills involved in listening is discussed.

Similarly, McNamara (1996) is a sourcebook on the development and validation of performance tests. McNamara (1996) introduced many-facet Rasch measurement (Linacre, 1994) as a useful method to capture the effect of external facets—most notably rater effects—on the measured performance of test takers. Relatedly, Bond and Fox (2007) guide readers through the general principles of the Rasch model and the various ways of applying it in their textbook. The importance of the Rasch model for test validation makes this accessible text oft-cited in studies concerned with test validity (e.g., Eckes and Grotjahn, 2006; Winke, 2011; Papageorgiou et al., 2012).

Another group of bursts in the cluster describe the then-current contexts of language assessment literacy (Taylor, 2009), frameworks (Fulcher, 2004), language tests after implementation (Alderson and Hamp-Lyons, 1996; Davison, 2007), and language for specific purposes (LSP, Douglas, 2000). In a call for the development of “assessment literacy” (Taylor, 2009) among applied linguists, Taylor described the state of the field of language assessment at that moment, looking at the types of practical knowledge needed and the scholarly work that offer them. This need for “assessment literacy” (Taylor, 2009) when implementing tests was already highlighted by Alderson and Hamp-Lyons (1996) some years before. Emphasizing the need to move beyond assumptions when hypothesizing about washback, Alderson and Hamp-Lyons (1996) observed and compared TOEFL and non-TOEFL classes taught by the same teachers in order to establish the presence of the oft-assumed washback effect of the TOEFL language tests. Davison (2007) takes a similar tack in looking at teachers' perception of the challenges in adapting to Hong Kong's shift to school-based assessment (SBA) of oral language skills. Although Davison (2007) and Alderson and Hamp-Lyons (1996) describe different tests, both sources highlight the importance of moving beyond theory and looking at implementation. That test development does not end at implementation is similarly highlighted by Fulcher (2004), who tackles the larger contexts surrounding the Common European Framework (CEF) in his critical historical overview of the development of said framework. Finally, Doughty's (2001) work on the assessment of LSP has become a major sourcebook in the field. Douglas's model of LSP ability drew inspiration from the communicative competence model of Canale and Swain (1980) and comprised language knowledge, strategic competence, and background knowledge.

#### Cluster 1: Rating (and Validation)

Moving from the global outlook on language assessment that largely characterizes Cluster 0, Cluster 1 narrows down on two related aspects of language testing: validation and rating. The unitary concept of validity (Messick, 1989), the socio-cognitive validity framework (Weir, 2005a), and the argument-based approach to validation (Kane, 2006, 2013) are the three main frameworks of validity featured in Cluster 1. The second major line of research in Cluster 1 is focused on improving rating scales. Fulcher (1996) proposed a data-driven approach to writing rating scales, coding transcripts from the ELTS oral examination to pinpoint “observed interruptions in fluency” (Fulcher, 1996, p. 216) present in candidates' speech. Using discriminant analysis, Fulcher (1996) linked linguistic descriptions to speaker performance, and at the same time, validating the rating scale produced. Iwashita et al. (2008) took a similar approach but expanded the range of measures beyond fluency with a more comprehensive set: grammatical accuracy and complexity, vocabulary, pronunciation, and fluency. Along the same idea, Fulcher et al. (2011) criticized the low richness of the descriptions generated from the measurement-driven approach and proposed Performance Decision Trees (PDTs), which are based on a non-linear scoring system that comprises yes/no decisions. In contrast, May (2011) took a different approach, using raters' perspectives to determine how raters would operationalize a rating scale and what features are salient to raters. Unlike the previous studies, however, the rating scale in May (2011) was for the paired speaking test. Mirroring the concerns about rating descriptors of speaking tasks, Knoch (2009) compared a new scale with more detailed, empirically developed descriptors with a pre-existing scale with less specific descriptors. Raters using the former scale reported higher rater reliability and better candidate discrimination. In a separate study, Knoch (2011) explained the features of diagnostic assessments of writing, stressing the uses and interpretations of rating scales.

With regards to the citing publications, papers describing the development of rating or scoring scales often cited the above publications, irrespective of what task the scale is for, resulting in the emergence of Cluster 1. For example, Banerjee et al.'s (2015) article focused the rating scale of writing assessment but discussed Fulcher (2003) and Fulcher et al. (2011). In addition, it is noted that rating scales are exclusively discussed with reference to the assessment of writing and speaking, with integrated tasks forming the nexus between these strands. Fulcher (2003) is the major publication of the speaking component of language assessment in this cluster, cited in studies focusing on speaking (Römer, 2017; van Batenburg et al., 2018) as well as meriting mention in studies on other topics like writing (Banerjee et al., 2015; Lallmamode et al., 2016). Akin to Fulcher (2003) for speaking, Weigle (2002) is a reference text on the subject of writing. It is cited in studies with a range of topics like integrated tasks (Plakans, 2008; Gebril, 2009; Plakans and Gebril, 2017), rubrics (Banerjee et al., 2015), validation (Lallmamode et al., 2016) and linguistic features of writing (Guo et al., 2013; Lu, 2017). Other citing papers focusing on writing assessment were Isbell (2017), Zhao (2017), Lam (2018), and Zheng and Yu (2019).

Measures of linguistic features in rater-mediated assessments have a significant importance in the cluster. Ortega's (2003) research synthesis quantified the effect size of syntactic complexity on assessed proficiency levels. More sophisticated ways of quantifying linguistic features have emerged since. A notable example is Coh-Metrix, a computational linguistic engine used to measure lexical sophistication, syntactic complexity, cohesion, and basic text information (Guo et al., 2013). McNamara et al. (2014) discussed the theoretical and practical implications of Coh-Metrix and provided an in-depth discussion of the textual features that Coh-Metrix measures. In a review article on syntactic complexity, Lu (2017) highlighted the increasing popularity of this tool. Coh-Metrix is used to operationalize and quantify linguistic and discourse features in writing, so as to predict scores (Banerjee et al., 2015; Wilson et al., 2017), test mode effect (Barkaoui and Knouzi, 2018).

#### Cluster 2: Test development (and dimensionality)

Cluster 2 is characterized by test development and dimensionality (see Supplemental Table 5). Publications in this cluster center around the development of tests (for teaching) (e.g., Oller, 1979; Henning, 1987; Hughes, 1989) and the implications of test scores, like Chen and Henning (1985), one of the initial works on bias. As well, a large part of the language test development process outlined in these publications include the interpretation and validation of test scores through item response theory (IRT) and Rasch models (Wright and Stone, 1979; Hambleton and Swaminathan, 1985; Henning, 1987). Rasch's (1960) pioneering monograph is the pillar upon which these publications stand. Citing articles are largely concerned with dimensionality (Lynch et al., 1988; McNamara, 1991) and validity (Lumley, 1993). From the publication dates, Cluster 2 seems reflective of prevailing concerns in the field specific to the 1980s and early 1990s.

#### Cluster 4: Rater Performance

As demonstrated in Supplemental Table 6, Cluster 4 concerns rating, which links it to Cluster 1. Chief concerns on variability in rating include raters' characteristics (Brown, 1995; Eckes, 2008), experience (Cumming, 1990; Lim, 2011) and biases (Lumley and McNamara, 1995) that affect rating performance, the effect of training (Weigle, 1994, 1998) and the processes by which the raters undergo while rating (Cumming et al., 2002; Lumley, 2002; Barkaoui, 2010). Citing articles largely mirror the same concerns (rater characteristics: Zhang and Elder, 2010; rater experience: Kim, 2015; rater training: Knoch et al., 2007; rating process: Wiseman, 2012; Winke and Lim, 2015), making this cluster a tightly focused one.

#### Cluster 5: Spoken Interaction

Cluster 5 looks at a specific aspect of assessing speaking: spoken interaction. Unlike Cluster 1 which also had a focus on assessing speaking, this cluster centers on a different group of bursts, thus its segregation: Brown (2003), Lazaraton (1996), Shohamy (1988), van Lier (1989) who explored the variation in the interactions between different candidates and testers during interviews. The social aspect of speaking calls into question validity and reliability in a strict sense, with implications for models of communicative ability, as Chalhoub-Deville (2003) highlighted. These developments in language assessment meant citing articles move beyond interviews to pair-tasks (O'Sullivan, 2002; Brooks, 2009; Davis, 2009), while maintaining similar concerns about reliability and validity (see Supplemental Table 7 for further information).

### Clusters in the General Journals Dataset

Table 9 demonstrates bursts in the influential clusters in the general journals dataset. The main clusters are discussed below.

**Table 9:** Selected cited publications (Bursts) in the general journals dataset.

**References**	**Burst strength**	**Frequency**	**Centrality**	**Sigma**	**Cluster ID**
Bachman (1990)	11.13	37	0.11	3.06	0
Oller (1979)	8.36	15	0.06	1.61	0
Henning (1987)	7.86	13	0.01	1.1	0
Wright and Stone (1979)	7.7	13	0.02	1.15	0
Halliday and Hasan (1976)	7.01	15	0.05	1.41	0
Hughes (1989)	5.7	9	0	1.03	0
Rasch (1960)	5.22	8	0.01	1.05	0
Chen and Henning (1985)	5.2	9	0.02	1.13	0
Bachman and Palmer (1982)	5.19	8	0.02	1.08	0
Hambleton and Swaminathan (1985)	4.78	8	0	1.01	0
Cohen (1988)	10.67	63	0.04	1.45	1
Swain (1995)	10.61	56	0.03	1.43	1
Ellis N. (2005)	10.3	56	0.03	1.33	1
Spada and Tomita (2010)	8.7	25	0.01	1.06	1
Pica (1994)	8.3	18	0.01	1.1	1
Lyster and Saito (2010)	8	20	0	1.03	1
Lyster and Ranta (1997)	7.48	38	0.02	1.18	1
Schmidt (1994)	7.2	18	0.01	1.08	1
Swain (1985)	7.08	42	0.03	1.2	1
Long (2007)	6.73	13	0	1.01	1
Goo (2012)	6.72	13	0	1.02	1
Harrington and Sawyer (1992)	6.61	19	0.01	1.04	1
Daneman and Carpenter (1980)	6.26	26	0.05	1.34	1
Ammar and Spada (2006)	6.03	28	0.01	1.04	1
Li (2010)	5.99	27	0	1.03	1
Doughty (2001)	5.96	14	0	1.01	1
(Ellis et al., 2006)	5.93	27	0.01	1.05	1
Schmidt (2001)	5.76	78	0.08	1.58	1
Ellis N. (2005)	5.69	11	0	1.02	1
Rebuschat (2013)	5.57	12	0	1	1
Sheen (2004)	5.41	15	0	1.01	1
(Ellis et al., 2001)	5.38	18	0.01	1.05	1
Gutiérrez (2013)	5.24	10	0	1.02	1
Lyster (1998)	5.24	10	0	1.01	1
Lyster (2004)	5.09	25	0.01	1.04	1
Long (1991)	5	15	0.02	1.09	1
Miyake and Friedman (1998)	4.8	13	0	1.01	1
Erlam (2005)	4.7	8	0	1	1
Mackey and Goo (2007)	4.66	8	0	1.01	1
Nation (1990)	11	33	0.05	1.67	2
Nation (2001)	8.95	67	0.03	1.36	2
Laufer and Hulstijn (2001)	7.1	23	0	1.03	2
Read (2000)	6.88	31	0.01	1.05	2
Nation (2006)	6.82	31	0.01	1.07	2
Read (2000)	6.74	18	0.01	1.06	2
Schmitt (2010)	6.68	20	0	1.01	2
(Godfroid et al., 2013)	6.5	14	0	1.02	2
Plonsky and Oswald (2014)	6.25	11	0	1.01	2
Laufer (1992)	6.12	16	0	1.03	2
Coxhead (2000)	6.02	31	0.04	1.24	2
Laufer and Ravenhorst-Kalovski (2010)	5.77	11	0	1.01	2
Nation (2013)	5.68	10	0	1	2
Waring and Takaki (2003)	5.58	14	0	1.01	2
Wray (2002)	5.56	13	0	1.01	2
Hulstijn (2003)	5.31	13	0	1.01	2
O'Malley and Chamot (1990)	5.16	11	0.01	1.05	2
Barr et al. (2013)	5.12	9	0	1.02	2
Boers et al. (2006)	5.05	11	0	1.01	2
Schmidt (2001)	4.72	9	0	1	2
Schmitt et al. (2001)	4.65	8	0	1	2
Canale and Swain (1980)	10.36	57	0.39	31.21	3
Alderson and Wall (1993)	6.15	11	0	1.03	3
Bachman and Palmer (1996)	4.82	27	0.02	1.1	3
Norris and Ortega (2009)	11.72	35	0.01	1.08	4
Norris and Ortega (2000)	9.81	48	0.03	1.37	4
Ellis (2003)	9.76	37	0.01	1.09	4
Skehan (1998)	8.59	65	0.08	1.91	4
Foster et al. (2000)	8.24	28	0.03	1.27	4
Skehan (2009)	8.02	24	0.01	1.07	4
Wolfe-Quintero et al. (1998)	7.01	21	0	1.02	4
Housen and Kuiken (2009)	6.65	13	0	1.02	4
Biber (1999)	6.38	16	0	1.03	4
Chandler (2003)	6.25	19	0.01	1.07	4
Levelt (1989)	6.2	12	0	1.02	4
Ellis (2009)	6.01	13	0	1.01	4
Vygotsky (1978)	5.68	10	0	1	4
Bates et al. (2015)	5.68	10	0	1	4
Larsen-Freeman (2006)	5.66	10	0	1	4
Ellis (2008)	5.65	20	0.01	1.03	4
Biber et al. (2011)	5.58	14	0	1.02	4
Kormos and Dénes (2004)	5.29	9	0	1	4
Ortega (2003)	5.18	13	0	1.02	4
Plonsky (2013)	4.78	12	0	1.02	4
Swain (2000)	4.74	12	0	1.01	4
Robinson (2005)	4.64	10	0	1	4
Dörnyei (2007)	4.64	10	0	1	4

#### Cluster 0: Test development (and dimensionality)

Cluster 0 in the General journals dataset overlapped in large part with Cluster 2 of the Core journals. Publications in Cluster 0 described the processes of test development (Oller, 1979; Wright and Stone, 1979; Henning, 1987; Hughes, 1989; Bachman, 1990). As with Cluster 2 (Core), there is a subfocus on IRT and Rasch models (Rasch, 1960; Wright and Stone, 1979; Hambleton and Swaminathan, 1985; Henning, 1987). Bachman (1990), Bachman and Palmer (1982), and Halliday and Hasan (1976) feature in this cluster but not in Cluster 2 (Core). There is a similar overlap in terms of the citing literature: 42% of the citing literature of the cluster overlaps with the citing literature of the Cluster 2 (Core), with little differences in central concerns of the articles (see Supplemental Table 8 for further information).

#### Cluster 1: Language Acquisition (Implicit vs. explicit)

Cluster 1 of the General journals dataset is a rather large cluster, which reflects the vastness of research into SLA. Long's (2007) book is one such attempt to elucidate on decades of theories and research. Other publications looked at specific theories like the output hypothesis (Swain, 1995), communicative competence (Swain, 1985) and the cognitive processes in language learning (Schmidt, 1994, 2001; Miyake and Friedman, 1998; Doughty, 2001). A recurrent theme in the theories of SLA is the dividing line between implicit and explicit language knowledge, as Ellis N. (2005) summarized. Research in the cluster similarly tackle the implicit and explicit divide in instruction (Ellis N., 2005; Erlam, 2005; Spada and Tomita, 2010). A subset of this is related to corrective feedback, where implicit feedback is often compared with explicit feedback (e.g., Ammar and Spada, 2006; Ellis et al., 2006). Along the same lines, Gutiérrez (2013) questions the validity of using grammaticality judgement tests to measure implicit and explicit knowledge (see Supplemental Table 9 for further information).

#### Cluster 2: Vocabulary Learning

Cluster 2 comprises of vocabulary learning research. General textbooks on theoretical aspects of vocabulary (Nation, 1990, 2001, 2013; O'Malley and Chamot, 1990; Schmitt, 2010) and Schmitt's (2008) review provide a deeper understanding of the crucial role of vocabulary in language learning, and in particular in incidental learning (Laufer and Hulstijn, 2001; Hulstijn, 2003; Godfroid et al., 2013). Efforts to find more efficient ways of learning vocabulary have led to the adoption of quantitative methods in research into vocabulary acquisition. Laufer (1992), Laufer and Ravenhorst-Kalovski (2010) and Nation (2006) sought the lexical threshold—the minimum number of words a learner needs for reading comprehension while the quantification of lexis allows for empirically-based vocabulary wordlists (Coxhead, 2000) and tests like the Vocabulary Levels Test (Schmitt et al., 2001). The use of formulaic sequences (Wray, 2002; Boers et al., 2006) is another off-shoot of this aspect of vocabulary learning. Read's (2000) text on assessing vocabulary remains a key piece of work, as it is in Cluster 0 of the Core journals. Finally, with the move toward quantitative methods, publications on relevant research methods such as effect size (Plonsky and Oswald, 2014) and linear mixed-effects models (Barr et al., 2013) gain importance in this cluster (see Supplemental Table 10 for further information).

#### Cluster 4: Measures of Language Complexity

Cluster 4 represent research on language complexity and its various measures. A dominant approach to measuring linguistic ability in this cluster is the measurement practices of complexity, accuracy, and fluency (CAF). In their review, Housen and Kuiken (2009) traced the historical developments and summarized the theoretical underpinnings and practical operationalization of the constructs, forming an important piece of work for research using CAF. Research in this cluster largely looked at the effect of methods of language teaching on one or more of the elements of CAF: for example, the effect of corrective feedback on accuracy and fluency (Chandler, 2003) and corrective feedback and the effect of planning on all three aspects in oral production (Ellis, 2009). Another line of research was to look at developments in complexity, accuracy, and/or fluency in students' language production (Ortega, 2003; Larsen-Freeman, 2006).

The CAF is not without its flaws, which are pointed out by Skehan (2009) and Norris and Ortega (2009). Norris and Ortega (2009) suggested that syntactic complexity should be measured multidimensionally and Biber et al. (2011), using corpus methods, suggested a new approach to syntactic complexity. As with Biber et al. (2011), another theme emerging from this cluster was the application of quantitative methods in language learning and teaching research (Bates et al., 2015). Methodological issues (Foster et al., 2000; Dörnyei, 2007; Plonsky, 2013) form another sub-cluster, as researchers attempt to come up with more precise ways of defining and measuring these constructs (see Supplemental Table 11 for further information).

### Second Aim: Measurement and Validation in the Core and General Journals

**Figure 3:**
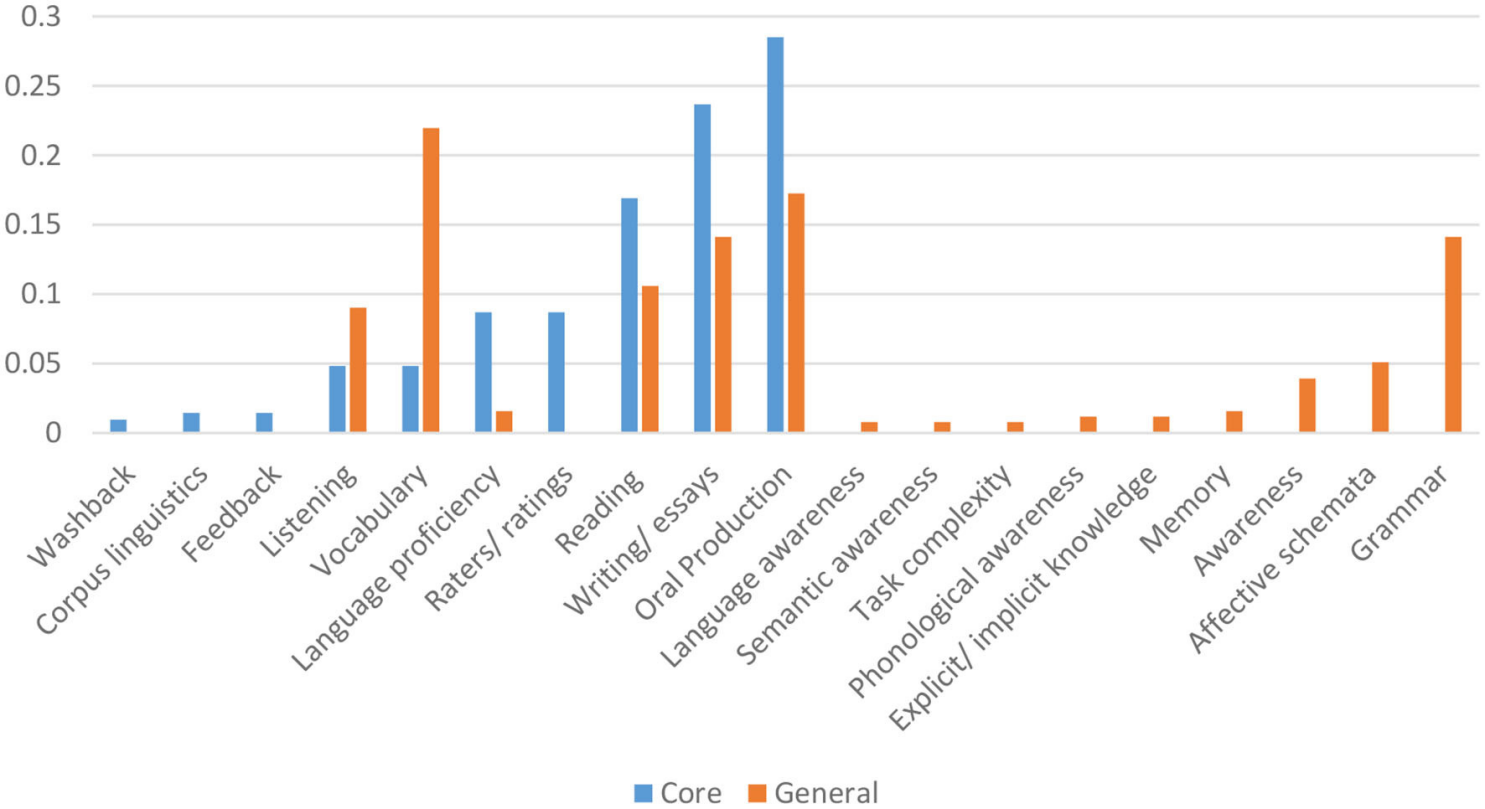
Comparison of domain specifications in the core and general journals.

**Figure 4:**
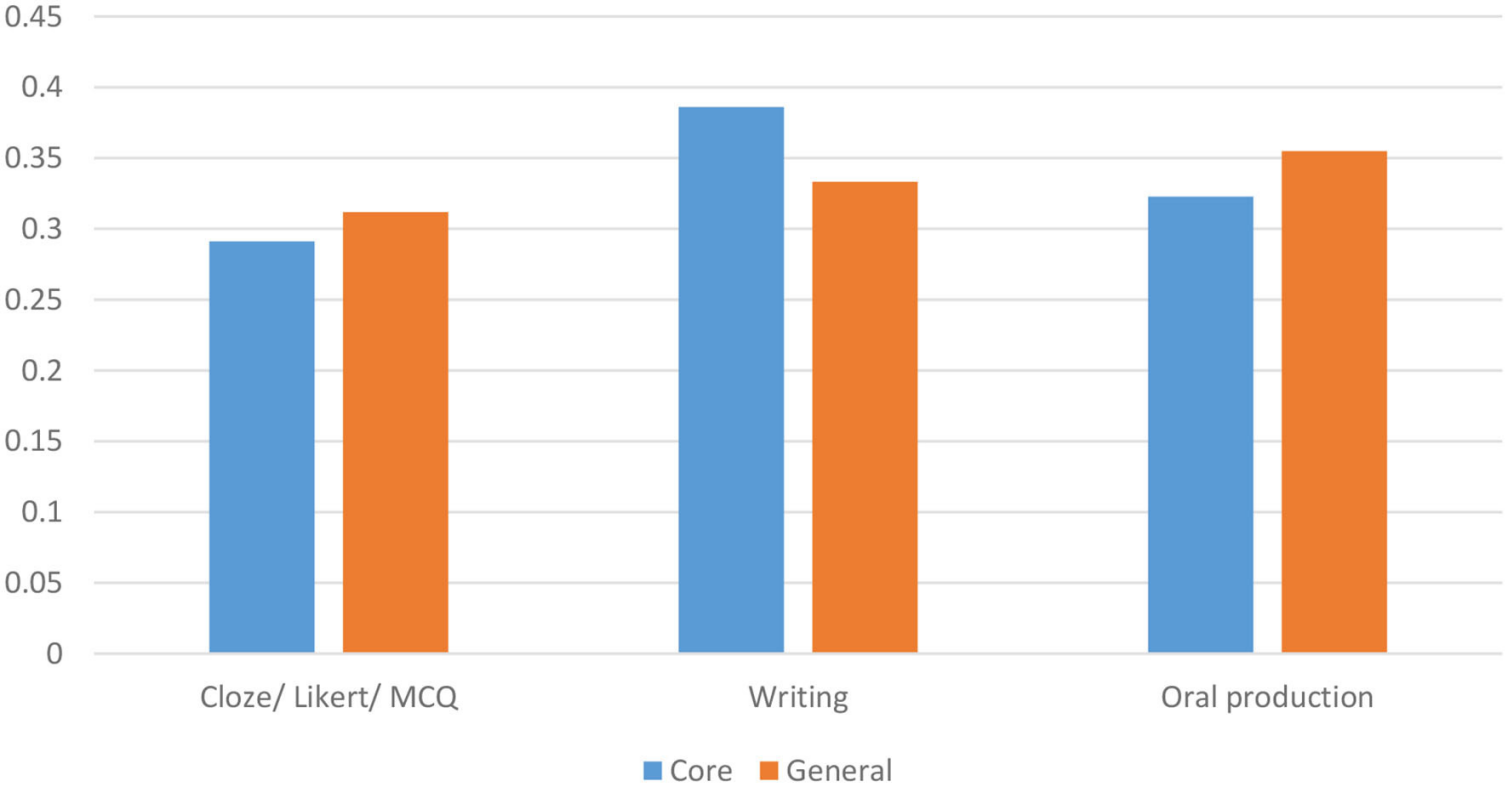
Comparison of construct operationalization in the core and general journals.

**Figure 5:**
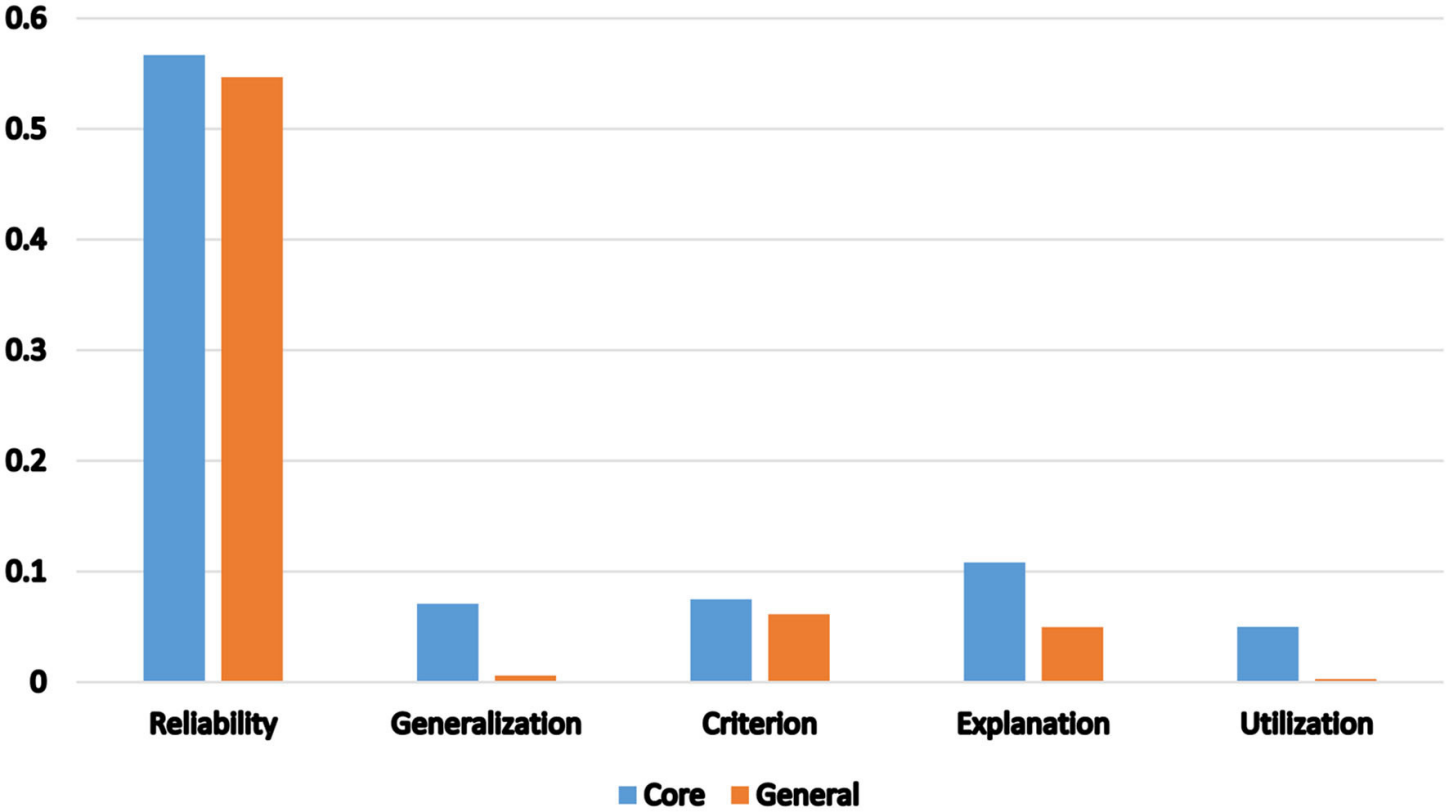
Comparison of measurement practices in the core and general journals.

The second aim of the study was to investigate measurement and validation practices in the published assessment research in the main clusters of the core and general journals. Figures 3–5 present visual comparisons in measurement and validation practices between the two datasets. Given the differing numbers in the two data sets, numbers presented in the histograms have been normalized for comparability (frequency of publications reporting the feature divided by the total number of papers). As demonstrated in Figure 3, studies in the general journals dataset covered a wider range of domain specifications, providing more coverage of more fine-grained domain specifications as compared to the core journals dataset. On the other hand, the four “basic” language skills—reading, writing, listening and speaking (listed here as Oral Production) were well-represented in both the general and core journals dataset, unsurprisingly. Cumulatively, reading, writing/essays, oral production dominate both the general journals and core journals datasets, with listening comparatively less so in both datasets. Of considerable interest is the predominance of vocabulary in the general journals dataset, far outstripping the four basic skills in the dataset.

In addition, as Figure 4 shows, the numbers of studies in both the core journals and general journals datasets that operationalized the constructs using Cloze/Likert/MCQ, Writing and Oral Production was fairly evenly matched. Writing is used most in the Core journals while Oral Production is used most in the General Journals. Finally, Figure 5 shows the importance placed on reliability by authors, in both datasets. In comparison, other measurement practices are scarcely given mention. Generalization and utilization had extremely poor showing in the general journals, in comparison to core journals, as the disparity between the four bars in Figure 5 shows.

### Limitations and Future Directions

The present study is not without limitations. As the focus of the study was to identify research clusters and bursts and the measurement and validation practices in language assessment research. However, the reasons why certain authors were co-cited by a large number of authors were not investigated. Merton (1968, 1988) and Small (2004) proposed two reasons for bursts in citations based on the sociology of science whereby the Matthew effect and the halo effect constitute possible contributors to the burstness of publications. First, Merton (1968, 1988) proposed that eminent authors often receive comparatively more credit from other authors than less known authors—Merton (1968, 1988) called this the Matthew Effect. This results in a widening lacuna between unknown and well-known authors (Merton, 1968, 1988) and in many cases the unfortunate invisibility of equally superior research published by unknown authors (Small, 2004). This is because citations function like “expert referral” and once they gain momentum, they “will increase the inequality of citations by focusing attention on a smaller number of selected sources, and widening the gap between symbolically rich and poor” (Small, 2004, p. 74). One way that this can be measured in future research is using power laws or similar mathematical functions to capture the trends in the data (Brzezinski, 2015). For example, a power law would fit a dataset of cited and citing publications wherein a large portion of the observed outcomes (citations) result from a small number of cited publications (Albarrán and Ruiz-Castillo, 2011). Albarrán et al. (2011, p. 395) provided compelling evidence from an impressively large dataset to support this phenomenon, concluding that “scientists make references that a few years later will translate into a highly skewed citation distribution crowned in many cases by a power law.”

In addition, the eminence of scholars or the reputation of journals where the work is published can make a significant contribution to their burstness—this is called the halo effect (Small, 2004). In a recent paper, Zhang and Poucke (2017) showed that journal impact factor has a significant impact on the citations that a paper received. Another study by Antoniou et al. (2015, p. 286) identified “study design, studies reporting design in the title, long articles, and studies with high number of references” as predictors of higher citation rates. To this list, we might add seniority and eminence of authors and the type of publication (textbooks vs. paper), as well as “negative citation, self-citation, and misattribution” (Small, 2004, p. 76). Future research should investigate whether these variables have a role in citation patterns and clusters that emerged in the present study.

While self-citation was not filtered out and may present a limitation of this study, self-citation can be legitimate and necessary to the continuity of the development of a line of research. In CiteSpace, to qualify as a citing article, the citations of the article must exceed a selection threshold, either by g-index, top N most cited per time slice, or other selection modes. Although this process does not prevent the selection of a self-cited reference, the selection is justifiable to a great extent. If a highly cited reference involves some or even all self-citations, then it behooves the analyst to establish the role of the reference in the literature. They should verify whether the high citations are due to inflated citations or if indeed, there is intellectual merit that justifies self-citation.

Another limitation of the study is that we did not include methodological journals such as “Journal of Educational Measurement” in the search, as indicated earlier. This was because we adopted a keyword search strategy in this study and the majority of the papers in methodological journals include the search keywords we used such as measurement and assessment, even though many of them are not relevant to language assessment. This would affect the quality and content of the clusters. We suggest future research can explore the relationship between language assessment and methodological journals through, for example, the dual-map overlay method which is available in CiteSpace. Similarly, technical reports and book chapters were not included in the datasets, as the former are not indexed in Scopus and coverage of Scopus of the latter is not as wide as its coverage of journal articles.

Finally, it should be noted that for a recent publication to become a burst, it will take at least 1 year as our present and past analyses show (Aryadoust and Ang, 2019). Therefore, the dynamics of the field under investigation can change in a few years, as new bursts and research clusters emerge and drag the direction of research to a different direction.

## Conclusion

The first aim of the study was to identify the main intellectual domains in language assessment research published in the core and general journals. We found that the primary focus of general journals was on vocabulary, oral proficiency, essay writing, grammar, and reading. The secondary focus was on affective schemata, awareness, memory, language proficiency, explicit vs. implicit language knowledge, language or semantic awareness, semantic complexity. By contrast, with the exception of language proficiency, this second area of focus was absent in the core generals. The focus of the core journals was more exclusively on reading and listening comprehension assessment (primary theme), facets of speaking and writing performance such as raters and (psychometric) validation (secondary theme), as well as feedback, corpus linguistics, and washback (tertiary theme). From this, it may be said the main preoccupation of researchers in SLA and language assessment was the assessment of reading, writing, and oral production, whereas assessment in SLA research additionally centered around vocabulary and grammar constructs. There were a number of areas that were underrepresented including affective schemata, awareness, memory, language proficiency, explicit vs. implicit language knowledge, language or semantic awareness, semantic complexity, feedback, corpus linguistics, and washback. These areas should be investigated with more rigor in future research.

In both datasets, several textbooks, editorials and review articles feature prominently in and/or across the clusters. The heavy presence of certain publications (like Bachman's) can be attributable to the importance of the scholar to the field. However, certain types of publications, like review articles, do tend to disproportionately get cited more often (Bennet et al., 2019) although precisely why this is the case is yet to be determined. Aksnes et al.'s (2019) cautioned on overreliance on bibliometric analysis ring true here as well. Thus, we have provided additional analyses on the statistics to complete the picture behind the numbers, inasmuch that is possible.

The second aim of the study was to describe measurement and validation practices in the two datasets. Collectively, the data and comparisons presented demonstrated strong evidence that the majority of citing papers did not carry out inference-based validation that was spelled out by Bachman and Palmer (2010), Kane (2006), or Messick (1989) in both core and general journals. In language assessment, Bachman (2005) and Bachman and Palmer (2010) stressed that an all-encompassing validation program is “important and *useful*” before an assessment can be put to any use (Bachman, 2005, p. 30, emphasis in original). However, the feasibility and heavy demands of a strong validity program remain an open question (see Haertel, 1999). Particularly, it seems impracticable to validate both the interpretations and uses of a language test/assessment before using the test for research purposes. The solution is Kane's (2006) less demanding approach which holds that test instruments should be validated for the claims made. Accordingly, it would not be expected that researchers provide any “validity” evidence containing all the validity inferences explicated above for every instrument. Some useful guidelines include the report of reliability (internal consistency and rater consistency), item difficulty and discrimination range, person ability range, as well as evidence that the test measures the purported constructs. In sum, in our view, the lack of reporting of evidence for the above-mentioned components in the majority of studies was because these were not applicable to the objectives and design of the studies and their assessment tools.

The preponderance of the use of open-ended (essay/oral performance), which engage more communicative skills as compared to discrete point/selected response testing (like MCQ or Cloze), shows a tendency toward communicative testing approaches in both datasets. As format effects have been found on L1 reading and L2 listening, and L2 listening under certain conditions (see In'nami and Koizumi, 2009), the popularity of the relatively more difficult open-ended questions have implications for language test developers that cannot be ignored. Given the effect of format on scores impacts the reliability of tests in making discriminations on language ability, and consequently, fairness, the popularity of one type of format in language testing should be re-evaluated, or at the very least, examined more closely.

Finally, the sustainability of the intellectual domains identified in this study depends on the needs of the language assessment community and other factors such as “influence” of the papers published in each cluster. If a topic is an established intellectual domain with influential authors (high burstness and betweenness centrality), it stands a higher chance of thriving and proliferating. However, the fate of intellectual domains that have not attracted the attention of authors with high bursts and betweenness centrality could be bleak—even though these clusters may discuss significant areas of inquiry. There is currently no profound understanding of the forces that shape the scope and direction of language assessment research. Significantly more research is needed to determine what motivates authors to select and investigate a topic, how thoroughly they cite past research, and what internal (within a field) and external (between fields) factors lead to the sustainability of a Research Topic.

## Data Availability Statement

Publicly available datasets were analyzed in this study. The datasets can be reproduced from Scopus using the search formula provided in the Appendix.

## Author Contributions

VA conceptualized the study, downloaded the data, conducted data analysis, contributed to writing the paper, and led the team. AZ and ML helped with the data analysis and coding, and contributed to writing the paper. CC contributed conceptually to data generation and analysis and suggested revisions. All authors contributed to the article and approved the submitted version.

## Conflict of Interest

The authors declare that the research was conducted in the absence of any commercial or financial relationships that could be construed as a potential conflict of interest. The handling editor is currently editing co-organizing a Research Topic with one of the author VA, and confirms the absence of any other collaboration.

## Footnotes

^1^We are grateful to one of the reviewers for suggesting this note.

^2^We did not include methodological journals such as ‘*Journal of Educational Measurement*’ in the search, as the majority of the papers in those journals include the search keywords, even though they are not relevant to language assessment.

^3^In DCA, some publications may not have a clear link with the rest of the publications in the dataset. These were not listed among the contributory publications to the major clusters that were visualized by CiteSpace in the presents study.

^4^CiteSpace, by default, shows the largest connected component. If a cluster does not appear in the largest connected component, this means it must appear in the second-largest connected component or other smaller components. The present study was limited to clusters within the largest connected component, which is a widely adopted strategy in network analysis.
