# Corrigendum: An Extensive Knowledge Mapping Review of Measurement and Validity in Language Assessment and SLA Research

**DOI:** 10.3389/fpsyg.2020.643027

**Published:** 2021-02-08

**Authors:** Vahid Aryadoust, Azrifah Zakaria, Mei Hui Lim, Chaomei Chen

**Affiliations:** ^1^National Institute of Education, Nanyang Technological University, Singapore, Singapore; ^2^Nanyang Technological University, Singapore, Singapore; ^3^College of Computing and Informatics, Drexel University, Philadelphia, PA, United States; ^4^Department of Information Science, Yonsei University, Seoul, South Korea

**Keywords:** document co-citation analysis, language assessment, measurement, review, Scientometrics, validity, visualization, Second language acquisition

In the original article, there were several errors throughout the text. The corrections made are detailed in the table below.

The authors apologize for these errors and state that they do not change the scientific conclusions of the article in any way. The original article has been updated.

**Table:** 

**Mistake made**	**Name of section/subsection**	**Paragraph number**	**Corrected text**
Lado (1961) and the book chapter of Carroll (1961),	INTRODUCTION	1 (p.1, line 113)	Lado's (1961) and the book chapter of Carroll's (1961),
Fulcher (n.d.)	INTRODUCTION	1 (p.2 line 133)	(Fulcher, n.d.)
in Bachman (1990) view	Second Aim	3 (p.3 line 271)	in Bachman's (1990) view
of Cronbach and Meehl (1955)	Second Aim	2 (p.3, l. 292)	of Cronbach and Meehl's (1955)
Messick	Second Aim	2 (p.3, l. 300)	Messick's
Kane (2006) frameworks	Second Aim	2 (p.3, l.301)	Kane's (2006) frameworks
Weir (2005b) socio-cognitive framework or	Second Aim	3 (p.3, l.306)	Weir's (2005b) socio-cognitive framework or
Borsboom and Mellenbergh (2007) test	Second Aim	3 (p.3, l.306-307)	Borsboom and Mellenbergh's (2007) test
Borsboom and Mellenbergh (2007) work	Second Aim	3 (p.3, l.308-309)	Borsboom and Mellenbergh's (2007) work
Weir (2005a) framework	Second Aim	3 (p.3, l.311)	Weir's (2005a) framework
target use domain (TLU)	Table 1, Row 1, Column 2	p.6, l. 574-575	target language use (TLU) domain
patterns on	Table 1, Row 4, Column 3	p. 6, l.650	patterns.
e.g., of which are too	First Aim: Characterizing the Detected Clusters; Cluster 0: Language assessment (and comprehension)	2 (p.13, l.1477)	examples of which are too
Carr (2006) study	First Aim: Characterizing the Detected Clusters; Cluster 0: Language assessment (and comprehension)	3 (p.16, l.1722)	Carr's (2006) study
Bachman and Palmer (1996) task characteristics	First Aim: Characterizing the Detected Clusters; Cluster 0: Language assessment (and comprehension)	3 (p.16, l.1723)	Bachman and Palmer's (1996) task characteristics
Carr (2006) investigation	First Aim: Characterizing the Detected Clusters; Cluster 0: Language assessment (and comprehension)	3 (p.16, l.1724)	Carr's (2006) investigation
Doughty (2001) work	First Aim: Characterizing the Detected Clusters; Cluster 0: Language assessment (and comprehension)	6 (p.16, l.1769)	Doughty's (2001) work
Banerjee et al. (2015) article	First Aim: Characterizing the Detected Clusters; Cluster 1: Rating (and Validation)	2 (p.16, l.1814)	Banerjee et al.'s (2015) article
Ortega (2003)	First Aim: Characterizing the Detected Clusters; Cluster 1: Rating (and Validation)	3 (p.17, l.1834)	Ortega's (2003)
Long (2007)'s	Cluster 1: Language Acquisition (Implicit vs. explicit)	1 (p. 17, l. 1922)	Long's (2007)
Aksnes et al. (2019) cautions	CONCLUSION	2 (p.22, l.2492)	Aksnes et al.'s (2019) cautions
DCA was conducted twice times	First Aim: Document Co-Citation Analysis (DCA)	1, (p. 4, l. 436)	DCA was conducted twice
Schmitt (2008) review	Cluster 2: Vocabulary Learning	1, (p.19, l. 2099)	Schmitt's (2008) review
Kane (2006)	Conclusion	3, (p.23, l.2510)	Kane's (2006)
