# Supplementary material for: An Extensive Knowledge Mapping Review of Measurement and Validity in Language Assessment and SLA Research
Source: Front Psychol. 2020 Sep 4;11:1941. doi: 10.3389/fpsyg.2020.01941 (PMC7500166; doi:10.3389/fpsyg.2020.01941)
Supplement: Supplementary file 1 [file Data_Sheet_1.pdf]

Aryadoust V, Azrifah Z, Lim MH, & Chen C (2020). An Extensive Knowledge Mapping Review of Measurement and Validity in Language Assessment and SLA Research. *Frontiers in Psychology, 11*:1941. doi: 10.3389/fpsyg.2020.01941

Supplemental Tables

Supplemental Table 1

Number (#) of Articles Published by the Top 20 Journals, Countries/Regions, and Institutions

Journals	Total # of papers
Language Testing	690
System	389
Language Learning	361
Assessing Writing	327
Applied Psycholinguistics	320
Modern Language Journal	287
English Language Teaching	237
TESOL Quarterly	192
Language Assessment Quarterly	187
Studies in Second Language Acquisition	173
Language Teaching Research	135
Language Testing in Asia	133
The RELC Journal	127
Computer Assisted Language Learning	120
Language Learning Journal	89
Recall	83
Language Awareness	78
Journal of Second Language Writing	73
Language Learning and Technology	72
English for Specific Purposes	64
Countries/Regions	
United States	1644
United Kingdom	448
Canada	334
Iran	241
Japan	233
Australia	207
China	181
Taiwan	150
Hong Kong	137
Netherlands	123
New Zealand	117
Spain	90
Israel	86
Germany	82
South Korea	78
Belgium	66
Turkey	59
Poland	51
Thailand	51
Saudi Arabia	50
Academic institutions	

Journals	Total # of papers
Educational Testing Service	99
University of Melbourne	92
Michigan State University	68
University of California, Los Angeles	65
Lancaster University	62
University of Auckland	56
University of Toronto	56
Georgia State University	55
University of Illinois at Urbana-Champaign	50
Pennsylvania State University	47
University of Hawaii at Manoa	47
Northern Arizona University	46
McGill University	45
Iowa State University	44
Victoria University of Wellington	41
The University of Hong Kong	41
University of Michigan, Ann Arbor	41
Concordia University	40
Universiteit van Amsterdam	38
Georgetown University	38

Supplemental Table 2

Publications with the Strongest Citation Bursts (Core and General)

	Burst strength	Beginning	End	Betweenness centrality	Sigma	Cluster ID
Bursts in core journals						
Bachman & Palmer (1996)	17.39	2009	2014	0.11	6.4	0
Bachman & Palmer (2010)	14.93	2016	2019	0.02	1.25	1
Bachman (1990)	11.77	2000	2004	0.35	32.79	3
Fulcher (2003)	11.54	2015	2019	0.01	1.10	1
Council of Europe (2001a)	11.17	2017	2019	0.01	1.11	1
Alderson et. al. (1995)	10.65	2010	2014	0.02	1.19	0
McNamara (1996)	9.30	2008	2012	0.02	1.19	4
American Educational Research Association et. al. (2014)	9.17	2016	2019	0.01	1.05	1
Weigle (2002)	9.05	2015	2019	0.05	1.6	1
Alderson (2000)	8.55	2006	2014	0.01	1.07	0
Cumming et. al. (2002)	8.48	2008	2015	0.01	1.1	4
Wright & Stone (1979)	8.10	1984	1994	0.05	1.48	2
Lumley (2002)	7.94	2007	2015	0.04	1.32	4
Skehan (1998)	7.90	2001	2003	0.01	1.1	3
Shohamy (2001)	7.84	2005	2013	0.01	1.1	0
Bursts in general journals						
Skehan (1998)	13.42	2004	2012	0.05	1.85	6
Bachman (1990)	12.15	1996	2002	0.05	1.81	3
Norris & Ortega (2009)	11.72	2014	2020	0.01	1.08	4
Nation (1990)	11.00	2005	2010	0.05	1.67	2
Cohen (1988)	10.67	2014	2020	0.04	1.45	1
Swain (1995)	10.61	2005	2014	0.03	1.43	1
Canale & Swain (1980)	10.36	1985	2000	0.39	31.21	3
Ellis (2005)	10.30	2011	2017	0.03	1.33	1
Norris & Ortega (2000)	9.81	2011	2017	0.03	1.37	4
Ellis (2003)	9.76	2011	2017	0.01	1.09	4
Nation (2001)	8.95	2011	2017	0.03	1.36	2
Spada & Tomita (2010)	8.70	2012	2017	0.01	1.06	1
Oller (1979)	8.36	1982	1995	0.06	1.61	0
Pica (1994)	8.30	2006	2011	0.01	1.10	1
Foster et. al. (2000)	8.24	2017	2020	0.03	1.27	4

Supplemental Table 3
Major Clusters in the Core Journal Dataset

Cluster ID	Size (cited papers)	Silhouette	Label (LLR)	Mean (Cite Year)	# of citers from the core journals
0	224	0.538	language assessment	1995	31(Language Testing)<br>25 (Language Assessment Quarterly)<br>2 (Language Testing in Asia)<br>1 (Assessing Writing)
1	221	0.544	interactional competence	2005	26 (Language Testing)<br>25 (Language Assessment Quarterly)<br>4 (Language Testing in Asia)<br>30 (Assessing Writing)
2	171	0.838	reading comprehension test	1981	21 (Language Testing)<br>0 (Language Assessment Quarterly)<br>0 (Language Testing in Asia)<br>0 (Assessing Writing)
3	161	0.753	task-based language assessment	1994	26 (Language Testing)<br>0 (Language Assessment Quarterly)<br>0 (Language Testing in Asia)<br>0 (Assessing Writing)
4	108	0.752	rater experience	1999	11 (Language Testing)<br>8 (Language Assessment Quarterly)<br>1 (Language Testing in Asia)<br>10 (Assessing Writing)
5	78	0.839	pair task performance	1993	18 (Language Testing)<br>1 (Language Assessment Quarterly)<br>0 (Language Testing in Asia)<br>0 (Assessing Writing)

Supplemental Table 4

Major Clusters in the General Journal Dataset

Cluster ID	Size (cited papers)	Silhouette	Label (LLR)	Mean (Cite Year)	# of citers from the general journals
2	133	0.568	incidental vocabulary learning	1998	1 (Iranian Journal of Language Teaching Research)<br>1 (Second Language Learning and Teaching)<br>2 (Journal of Second Language Writing)<br>2 (Language Learning Journal)<br>2 (RELC Journal)<br>2 (TESOL Quarterly)<br>3 (English Language Teaching)<br>3 (Language Learning and Technology)<br>3 (Language Testing)<br>3 (ReCALL)<br>4 (Applied Psycholinguistics)<br>5 (Language Awareness)<br>6 (Computer Assisted Language Learning)<br>8 (Language Learning)<br>8 (Modern Language Journal)<br>8 (Studies in Second Language Acquisition)<br>10 (System)<br>13 (Language Teaching Research)
1	130	0.656	corrective feedback	2001	1 (Journal of Second Language Writing)<br>1 (Language Learning and Development)<br>1 (Language Learning Journal)<br>1 (TESOL Quarterly)<br>13 (Language Learning)<br>2 (Computer Assisted Language Learning)<br>2 (Language Learning and Technology)<br>2 (Second Language Learning and Teaching)<br>3 (Applied Psycholinguistics)<br>3 (Language Awareness)<br>4 (System)<br>6 (Language Teaching Research)<br>7 (Modern Language Journal)<br>20 (Studies in Second Language Acquisition)
3	98	0.771	language testing	1989	10 (System)<br>8 (Language Testing)

Cluster ID	Size (cited papers)	Silhouette	Label (LLR)	Mean (Cite Year)	# of citers from the general journals
0	96	0.86	foreign language aptitude	1980	4 (Second Language Learning and Teaching)<br>4 (TESOL Quarterly)<br>4 (Language Learning)<br>3 (Studies in Second Language Acquisition)<br>2 (English for Specific Purposes)<br>2 (Language Learning and Technology)<br>1 (Journal of Second Language Writing)<br>1 (RELC Journal)<br>1 (Language Teaching Research)<br>1 (Modern Language Journal)<br>26 (Language Testing)<br>6 (TESOL Quarterly)<br>4 (Language Learning)<br>1 (RELC Journal)<br>1 (English for Specific Purposes)<br>1 (Computer Assisted Language Learning)<br>2 (Iranian Journal of Language Teaching Research)<br>2 (Language Teaching Research)
4	96	0.698	syntactic complexity	2003	3 (Language Learning)<br>3 (TESOL Quarterly)<br>5 (Modern Language Journal)<br>5 (Modern Language Journal)<br>9 (System)<br>19 (Journal of Second Language Writing)<br>1 (Applied Psycholinguistics)<br>1 (Computer Assisted Language Learning)<br>1 (Language Awareness)
6	57	0.806	narrative task performance	1992	1 (Language Teaching Research)<br>1 (TESOL Quarterly)<br>3 (Modern Language Journal)<br>3 (System)<br>6 (Language Learning)<br>7 (Studies in Second Language Acquisition)
8	45	0.936	second language	1986	6 (Language Learning)<br>2 (System)<br>1 (Modern Language Journal)<br>1 (Studies in Second Language Acquisition)

Cluster ID	Size (cited papers)	Silhouette	Label (LLR)	Mean (Cite Year)	# of citers from the general journals
5	42	0.948	phonological awareness	1991	1 (Language Learning and Technology)<br>1 (Language Awareness)<br>13 (Applied Psycholinguistics)<br>1 (Language Learning)<br>1 (RELC Journal)
11	42	0.839	Japanese relative clauses	1995	5 (Studies in Second Language Acquisition)<br>4 (Language Awareness)<br>2 (System)<br>1 (English Language Teaching)<br>1 (The Modern Language Journal)

Supplemental Table 5

Citing and Cited Publications in Cluster 2 of the Core Journals

Cluster	Publication	Citing	Cited (bursts)	Focus area 1	Focus area 2
2	Wright & Stone (1979)		X	Test development	The Rasch models
2	Henning (1987)		X	Test development	The Rasch models
2	Oller (1979)		X	Test development	
2	Rasch (1960)		X	The Rasch models	
2	Hambleton & Swaminathan (1985)		X	Item response theory	
2	Hughes (1989)		X	Test development	Language learning
2	McNamara (1990)		X	Test development	The Rasch models
2	Chen & Henning (1985)		X	Test fairness	
2	Lynch et al. (1988)	X		Person dimensionality	The Rasch models
2	McNamara (1991)	X		The Rasch models	Dimensionality

Supplemental Table 6
Citing and Cited Publications in Cluster 4 of the Core Journals

Cluster	Publication	Citing	Cited (bursts)	Focus area 1	Focus area 2
4	Cumming, Kantor & Powers (2002)		X	Rating process	
4	Lumley (2002)		X	Rating process	
4	Cumming (1990)		X	Rater characteristics	Rating process
4	Eckes (2008)		X	Rater characteristics	Rating process
4	Lumley & McNamara (1995)		X	Rater characteristics	Rater training
4	Weigle (1998)		X	Rater training	
4	Weigle (1994)		X	Rater training	
4	Brown (1995)		X	Rater characteristics	
4	Lim (2011)		X	Rater characteristics	Rating variability
4	Barkaoui (2010)	X	X	Rating process	Rating variability
4	Wiseman (2012)	X		Rating process	Rater characteristics
4	Winke & Lim (2015)	X		Rating process	Eye-tracking
4	Knoch, Read & von Randow (2007)	X		Rater training	
4	Zhang & Elder (2010)	X		Rater characteristics	
4	Kim (2015)	X		Rater characteristics	

Supplemental Table 7

Citing and Cited Publications in Cluster 5 of the Core Journals

Cluster	Publication	Citing	Cited (bursts)	Focus area 1	Focus area 2
5	Brown (2003)		X	Speaking (Interaction)	Rater variation
5	van Lier (1989)		X	Oral interview	Validity
5	Lazaraton (1996)		X	Speaking (interaction)	-
5	Messick (1996)		X	Validity	Washback
5	Chalhoub-Deville (2003)		X	Review of spoken interaction	-
5	Shohamy (1988)		X	Speaking (Interaction)	Framework
5	O'Sullivan (2002)	X		Pair-task performance	Interlocutor familiarity
5	Brooks (2009)	X		Pair-task performance	Testing format
5	Davis (2009)	X		Pair-task performance	Interlocutor proficiency

Supplemental Table 8
Cited Publications in Cluster 0 of the General Journals

Cluster	Publication	Citing	Cited (bursts)	Focus area 1	Focus area 2
0	Bachman (1990)		X	Test development	Facets of test development
0	Oller (1979)		X	Test development	Language learning
0	Henning (1987)		X	Test development (IRT and Rasch models)	Language learning
0	Wright & Stone (1979)		X	Test development	IRT and Rasch models
0	Halliday & Hasan (1976)		X	Cohesion	Language
0	Hughes (1989)		X	Test development	Language learning
0	Rasch (1960)		X	IRT and Rasch models	
0	Chen & Henning (1985)		X	Test fairness	
0	Bachman & Palmer (1982)		X	Construct validation	Communicative competence
0	Hambleton & Swaminathan (1985)		X	Item response theory	

Note: due to the space constraints, citing publications are not displayed in the table.

Supplemental Table 9

Cited Publications in Cluster 1 of the General Journals

Cluster	Publication	Citing	Cited (bursts)	Focus area 1	Focus area 2
1	Cohen (1988)		X	Research Methods	Statistics
1	Swain (1995)		X	Output hypothesis	
1	Ellis, R. (2005)		X	Explicit vs implicit instruction	
1	Spada & Tomita (2010)		X	Meta-analysis of explicit vs implicit instruction	
1	Pica (1994)		X	Social interaction	Input
1	Lyster & Saito (2010)		X	Meta-analysis of oral corrective feedback	
1	Lyster & Ranta (1997)		X	Corrective feedback	Feedback type effectiveness
1	Schmidt (1994)		X	SLA Theories	Cognitive processes in language learning
1	Swain (1985)		X	SLA Theories	Communicative Competence
1	Long (2007)		X	SLA theories	
1	Goo (2012)		X	Corrective feedback	Working memory
1	Harrington & Sawyer (1992)		X	Working memory	Reading
1	Daneman & Carpenter (1980)		X	Working memory	Reading
1	Ammar & Spada (2006)		X	Corrective feedback	Recasts and prompts
1	Li (2010)		X	Meta-analysis on corrective feedback	
1	Doughty (2001)		X	Focus on Form	Cognitive processes in language learning
1	Ellis, Loewen & Erlam (2006)		X	Corrective feedback	Recasts and metalinguistic
1	Schmidt (2001)		X	Attention	Cognitive processes in language learning
1	Ellis, N. (2005)		X	SLA theories	Implicit and Explicit Knowledge
1	Rebuschat (2013)		X	Research Methods	Implicit and Explicit Knowledge
1	Sheen (2004)		X	Corrective feedback	Communicative ESL teaching
1	Ellis, Basturkmen & Loewen (2001)		X	Focus on Form	Communicative ESL teaching
1	Gutiérrez (2013)		X	Construct Validity	Implicit and Explicit Knowledge
1	Lyster (1998)		X	Corrective feedback	Recasts

Cluster	Publication	Citing	Cited (bursts)	Focus area 1	Focus area 2
1	Lyster (2004)		X	Corrective feedback	Focus on Form
1	Miyake & Friedman (1998)		X	Working memory	
1	Erlam (2005)		X	Explicit vs implicit instruction	
1	Mackey & Goo (2007)		X	Meta-analysis on interaction	

Note: due to the space constraints, citing publications are not displayed in the table.

Supplemental Table 10
Cited Publications in Cluster 2 of the General Journals

Cluster	Publication	Citing	Cited (bursts)	Focus area 1	Focus area 2
2	Nation (1990)		X	Vocabulary Learning	
2	Nation (2001)		X	Vocabulary Learning	
2	Laufer & Hulstijn (2001)		X	Review of incidental vocabulary acquisition	
2	Read (2000)		X	Vocabulary	Assessment
2	Nation (2006)		X	Lexical threshold	Corpus methods
2	Schmitt (2010)		X	Research methods	Vocabulary
2	Godfroid, Boers & Housen (2013)		X	Incidental Vocabulary Acquisition	Attention
2	Plonsky & Oswald (2014)		X	Research methods	Effect size
2	Laufer (1992)		X	Lexical threshold	Corpus methods
2	Coxhead (2000)		X	Lexical frequency	Corpus methods
2	Laufer & Ravenhorst-Kalovski (2010)		X	Lexical threshold	Corpus methods
2	Nation (2013)		X	Vocabulary	Learning
2	Waring & Takaki (2003)		X	Vocabulary learning	Rate of acquisition
2	Wray (2002)		X	Formulaic sequences	Theoretical model
2	Hulstijn (2003)		X	Vocabulary acquisition	Incidental vs. intentional
2	O'Malley & Chamot (1990)		X	Vocabulary Learning	-
2	Barr et al. (2013)		X	Research methods	Linear mixed-effects models
2	Boers et al. (2006)		X	Vocabulary learning	Formulaic sequences
2	Schmitt, Schmitt, & Clapham (2001)		X	Test development (Vocabulary Levels Test)	Validity
2	Schmitt (2008)		X	Review of vocabulary learning	

Note: due to the space constraints, citing publications are not displayed in the table.

Supplemental Table 11

Cited Publications in Cluster 4 of the General Journals

Cluster	Publication	Citing	Cited (bursts)	Focus area 1	Focus area 2
4	Norris & Ortega (2009)		X	Complexity, accuracy, and fluency (CAF) framework	
4	Norris & Ortega (2000)		X	Meta-analysis of L2 instruction	
4	Ellis (2003)		X	Task-based language learning and teaching	
4	Skehan (1998)		X	Cognitive processes in language learning	Task-based language learning and teaching
4	Foster, Tonkyn & Wigglesworth (2000)		X	Review of measures of speaking	
4	Skehan (2009)		X	Complexity, accuracy, and fluency (CAF) framework	
4	Wolfe-Quintero, Inagaki & Kim (1998)		X	Review of measures of writing	
4	Housen & Kuiken (2009)		X	Review of CAF	
4	Biber et al. (1999)		X	Grammar	Speaking and Writing
4	Chandler (2003)		X	Corrective feedback	Accuracy and Fluency (Writing)
4	Levelt (1989)		X	Cognitive Processes	Speaking
4	Ellis (2009)		X	Complexity, accuracy, and fluency (CAF)	Speaking
4	Vygotsky (1978)		X	Mind and society	
4	Bates, Mächler, Bolker, & Walker (2015)		X	Research methods (Linear mixed-effects models)	
4	Larsen-Freeman (2006)		X	CAF	Speaking and Writing
4	Ellis (2008)		X	SLA theories	SLA teaching
4	Biber, Gray, & Poonpon (2011)		X	Syntactic Complexity	Speaking and Writing
4	Kormos & Dénes (2004).		X	Fluency	Speaking
4	Ortega (2003)		X	Syntactic Complexity	Writing

Cluster	Publication	Citing	Cited (bursts)	Focus area 1	Focus area 2
4	Plonsky (2013)		X	Meta-analysis on quantitative SLA research	Research methods
4	Swain (2000)		X	The output hypothesis	Language acquisition
4	Robinson (2005)		X	Cognitive processes in language learning	Task-based language learning and teaching
4	Dörnyei (2007)		X	Research Methods	

Note: due to the space constraints, citing publications are not displayed in the table.

Appendix

Core search

(SRCTITLE ("Language Testing") OR SRCTITLE ("Language Assessment Quarterly") OR SRCTITLE ("Language Testing in Asia") OR SRCTITLE ("Assessing Writing")) AND (LIMIT-TO (EXACTSRCTITLE , "Language Testing") OR LIMIT-TO (EXACTSRCTITLE , "Assessing Writing") OR LIMIT-TO (EXACTSRCTITLE , "Language Assessment Quarterly") OR LIMIT-TO (EXACTSRCTITLE , "Language Testing In Asia"))

General search

((SRCTITLE ("Applied Linguistics") OR SRCTITLE ("Studies in second language acquisition") OR SRCTITLE ("TESOL Quarterly") OR SRCTITLE ("Language awareness") OR SRCTITLE ("modern language journal") OR SRCTITLE ("English Language Teaching") OR SRCTITLE ("language learning") OR SRCTITLE ("Language Teaching Research") OR SRCTITLE ("computer assisted language learning") OR SRCTITLE ("Journal of Second Language Writing") OR SRCTITLE ("ReCALL") OR SRCTITLE ("English for Specific Purposes") OR SRCTITLE ("Language Learning and Technology") OR SRCTITLE ("Applied Psycholinguistics") OR SRCTITLE ("RELC Journal") OR SRCTITLE ("System"))) AND (test OR assess OR rate OR measure) AND (LIMIT-TO (DOCTYPE , "ar")) AND (LIMIT-TO (SUBJAREA , "SOCI") OR LIMIT-TO (SUBJAREA , "ARTS")) AND (LIMIT-TO (EXACTSRCTITLE , "Modern Language Journal") OR LIMIT-TO (EXACTSRCTITLE , "System") OR LIMIT-TO (EXACTSRCTITLE , "Language Learning") OR LIMIT-TO (EXACTSRCTITLE , "Tesol Quarterly") OR LIMIT-TO (EXACTSRCTITLE , "Applied Psycholinguistics") OR LIMIT-TO (EXACTSRCTITLE , "Relc Journal") OR LIMIT-TO (EXACTSRCTITLE , "Applied Linguistics") OR LIMIT-TO (EXACTSRCTITLE , "English Language Teaching") OR LIMIT-TO (EXACTSRCTITLE , "English For Specific Purposes") OR LIMIT-TO (EXACTSRCTITLE , "Studies In Second Language Acquisition") OR LIMIT-TO (EXACTSRCTITLE , "Computer Assisted Language Learning") OR LIMIT-TO (EXACTSRCTITLE , "Recall") OR LIMIT-TO (EXACTSRCTITLE , "Language Awareness") OR LIMIT-TO (EXACTSRCTITLE , "Journal Of Second Language Writing") OR LIMIT-TO (EXACTSRCTITLE , "Language Learning And Technology") OR LIMIT-TO (EXACTSRCTITLE , "Applied Linguistics Review") OR LIMIT-TO (EXACTSRCTITLE , "Annual Review Of Applied Linguistics"))

Combined search

((SRCTITLE ("Language Testing") OR SRCTITLE ("Language Assessment Quarterly") OR SRCTITLE ("Language Testing in Asia") OR SRCTITLE ("Assessing Writing"))) OR (((SRCTITLE ("Applied Linguistics") OR SRCTITLE ("Studies in second language acquisition") OR SRCTITLE ("TESOL Quarterly") OR SRCTITLE ("Language awareness") OR SRCTITLE ("modern language journal") OR SRCTITLE ("English Language Teaching") OR SRCTITLE ("language learning") OR SRCTITLE ("Language Teaching Research") OR SRCTITLE ("computer assisted language learning") OR SRCTITLE ("Journal of Second Language Writing") OR SRCTITLE ("ReCALL") OR SRCTITLE ("English for Specific Purposes") OR SRCTITLE ("Language Learning and Technology") OR SRCTITLE ("Applied Psycholinguistics") OR SRCTITLE ("RELC Journal") OR SRCTITLE ("System")))) AND (test OR assess OR rate OR measure)) AND (LIMIT-TO (SUBJAREA , "SOCI") OR LIMIT-TO (SUBJAREA , "ARTS")) AND (LIMIT-TO (DOCTYPE , "ar")) AND (LIMIT-TO (EXACTSRCTITLE , "Language Testing") OR LIMIT-TO (EXACTSRCTITLE , "Assessing Writing") OR LIMIT-TO (EXACTSRCTITLE , "Language Assessment Quarterly") OR LIMIT-TO (EXACTSRCTITLE , "Language Testing In Asia") OR LIMIT-TO (EXACTSRCTITLE , "Modern Language Journal") OR LIMIT-TO (EXACTSRCTITLE , "System") OR LIMIT-TO (EXACTSRCTITLE , "Language Learning") OR LIMIT-TO (EXACTSRCTITLE , "Tesol Quarterly") OR LIMIT-TO (EXACTSRCTITLE , "Applied Psycholinguistics") OR LIMIT-TO (EXACTSRCTITLE , "Relc Journal") OR LIMIT-TO (EXACTSRCTITLE , "Applied Linguistics") OR LIMIT-TO (EXACTSRCTITLE , "English Language Teaching") OR LIMIT-TO (EXACTSRCTITLE , "English For Specific Purposes") OR LIMIT-TO (EXACTSRCTITLE , "Studies In Second Language Acquisition") OR LIMIT-TO (EXACTSRCTITLE , "Computer Assisted Language Learning") OR LIMIT-TO (EXACTSRCTITLE , "Recall") OR LIMIT-TO (EXACTSRCTITLE , "Language Awareness") OR LIMIT-TO (EXACTSRCTITLE , "Journal Of Second Language Writing") OR LIMIT-TO (EXACTSRCTITLE , "Language Learning And Technology") OR LIMIT-TO (EXACTSRCTITLE , "Applied Linguistics Review") OR LIMIT-TO (EXACTSRCTITLE , "Annual Review Of Applied Linguistics"))
